# Genetic Variation and Association Mapping of Seed-Related Traits in Cultivated Peanut (*Arachis hypogaea* L.) Using Single-Locus Simple Sequence Repeat Markers

**DOI:** 10.3389/fpls.2017.02105

**Published:** 2017-12-11

**Authors:** Jiaojiao Zhao, Li Huang, Xiaoping Ren, Manish K. Pandey, Bei Wu, Yuning Chen, Xiaojing Zhou, Weigang Chen, Youlin Xia, Zeqing Li, Huaiyong Luo, Yong Lei, Rajeev K. Varshney, Boshou Liao, Huifang Jiang

**Affiliations:** ^1^Key Laboratory of Biology and Genetic Improvement of Oil Crops, Ministry of Agriculture, Oil Crops Research Institute of the Chinese Academy of Agricultural Sciences, Wuhan, China; ^2^International Crops Research Institute for the Semi-Arid Tropics, Hyderabad, India; ^3^Nanchong Academy of Agricultural Sciences, Nanchong, China; ^4^Shanghai Igenebank Biotechnology Company Limited, Shanghai, China

**Keywords:** association mapping, peanut, seed-related traits, single-locus SSR, linkage disequilibrium

## Abstract

Cultivated peanut (*Arachis hypogaea* L.) is an allotetraploid (AABB, 2*n* = 4*x* = 40), valued for its edible oil and digestible protein. Seed size and weight are important agronomical traits significantly influence the yield and nutritional composition of peanut. However, the genetic basis of seed-related traits remains ambiguous. Association mapping is a powerful approach for quickly and efficiently exploring the genetic basis of important traits in plants. In this study, a total of 104 peanut accessions were used to identify molecular markers associated with seed-related traits using 554 single-locus simple sequence repeat (SSR) markers. Most of the accessions had no or weak relationship in the peanut panel. The linkage disequilibrium (LD) decayed with the genetic distance of 1cM at the genome level and the LD of B subgenome decayed faster than that of the A subgenome. Large phenotypic variation was observed for four seed-related traits in the association panel. Using mixed linear model with population structure and kinship, a total of 30 significant SSR markers were detected to be associated with four seed-related traits (*P* < 1.81 × 10^-3^) in different environments, which explained 11.22–32.30% of the phenotypic variation for each trait. The marker AHGA44686 was simultaneously and repeatedly associated with seed length and hundred-seed weight in multiple environments with large phenotypic variance (26.23 ∼ 32.30%). The favorable alleles of associated markers for each seed-related trait and the optimal combination of favorable alleles of associated markers were identified to significantly enhance trait performance, revealing a potential of utilization of these associated markers in peanut breeding program.

## Introduction

Cultivated peanut (*Arachis hypogaea* L.), an excellent source of edible oil and proteins, is one of the most important oilseed crops in the world. Cultivated peanut is an allotetraploid species (AABB, 2*n* = 40), that evolved from a single natural hybridization between two diploid progenitor species, *A. duranensis* (AA, 2*n* = 20) and *A. ipaensis* (BB, 2*n* = 20), followed by chromosome duplication (Kochert et al., 1996; Bertioli et al., 2016). It is widely cultivated in more than 100 countries due to its key role in human nutrition, especially in Asia and Africa, in which the production of peanut accounted for approximately 90% in the global annual production. With the increasing demand for peanut in human life, the global area harvested raised from 22.8 Mha in 2012 to 25.7 Mha in 2014^1^. However, the peanut production is difficult to satisfy mass consumption. Thus there is great potential to enhance peanut global production through increasing the plant productivity.

Yield is a complex and quantitative trait, which is directly and indirectly influenced by multiple components agronomic traits. Generally, seed-related traits including seed length (SL), seed width (SW), ratio of SL to width (SL/W) and hundred-seed weight (HSW) significantly and directly impact the peanut yield, especially the plant productivity. Furthermore, the seed-related traits also have effects on the nutrients composition (Prathiba and Reddy, 1994) and flavor (Pattee et al., 2002) in addition to the desirable seed features meeting the industry preferences. Therefore, there is a need for understanding the genetic basis of seed-related traits and identification of genetic factors would help in improving these traits using marker-assisted selection. Linkage mapping based on segregating population derived from bi-parental crossing is a routine approach for dissecting the genetic basis of target traits including seed-related traits in peanut. A number of quantitative trait loci (QTLs) for SL, SW and seed weight were detected using linkage mapping (Shirasawa et al., 2012; Huang L. et al., 2015; Chen et al., 2016). However, the resolution of linkage mapping in peanut was relatively low due to the limited polymorphism between two parents. Further, the development of segregating population especially recombinant inbred line (RIL) was very laborious and time-consuming. In addition, the complexity of peanut genome has further slowed down the process of fine mapping and candidate gene discovery in the QTL region identified for seed-related traits in earlier studies in peanut.

In recent years, association mapping based on extensive historical recombination in diversely natural population has been an efficient approach to identify QTL for important agronomic traits and successfully conducted in model and non-model crops (Cai et al., 2014; Huang X. et al., 2015; Lquira et al., 2015; Xu et al., 2015; Xiao et al., 2016; Si et al., 2016). With the rapid development of high-throughput sequencing technology, single nucleotide polymorphism (SNP) markers have been popular molecular markers used in association mapping because of wide distribution and a large amount in genome. Although the high-quality reference genome sequences of two wild diploid ancestors of cultivated peanut have been published (Bertioli et al., 2016), wide-range utilization of SNP markers are limited in peanut due to the complexity of polyploidy genome structure and large genome size (2.7 ∼ 2.8 G). Thus simple sequence repeat (SSR) markers which feature multi-allelic nature, codominant heritability and genome-wide dispersal are the most preferred genetic markers in association mapping in peanut. Previously, ‘Reference Set’ developed by ICRISAT (Pandey et al., 2014) was conducted genome-wide association studies (GWAS) using no more than 150 SSR markers leading to identification of a small number of marker-trait associations (MTAs) for seed-related traits.

Generally, the SSR markers in peanut could result in multiple loci because of the high homology between A and B subgenome. The application of multi-locus SSR markers in genetic studies could lead to inaccuracy of assessment in number of alleles, allele frequency and polymorphism information content (PIC) in each marker (Chen et al., 2008; Li et al., 2012). Furthermore, the multi-locus SSR markers are unsuitable to evaluate the population structure and linkage disequilibrium (LD) because of ambiguous genotyping in natural population of allopolyploid. Thus, the single-locus SSR markers are more fit to association mapping in allopolyploid (Comadran et al., 2009; Jin et al., 2010).

Although an association study for seed-related traits has already been carried out in the peanut mini-core collection (Jiang et al., 2014) that encompass the 99 accessions of mini–mini core collection used in this study, there were two advantages that lacked in the previous study. Firstly, five additional accessions that were parental lines of three segregating populations were added in this study to contribute to the QTLs identified using association mapping and linkage mapping in our further studies. Moreover, the application of single-locus markers could benefit the accuracy of MTAs. The association analysis in the previous study (Jiang et al., 2014) was conducted using alleles of 109 SSR markers and each polymorphic allele was obtained according to the presence or absence of the amplification due to the limitation of the number of SSR markers developed at that time. Currently, large efforts have been made to develop the SSR markers and thousands of SSR markers have been developed in peanut using genomic survey sequencing. Thus, the development of SSR markers promoted the identification of the single-locus markers and the use of that in MTAs in this study.

In order to efficiently utilize germplasm resources in peanut, we conducted GWAS analysis using the genotyping data and multi-environment phenotyping data on four seed-related traits in the Chinese mini–mini core collection and five diverse peanut accessions. Therefore, the objectives of this study were (a) to assess the genetic diversity and population structure of the peanut mini–mini core panel, (b) to identify SSR markers associated with seed-related traits, and (c) to evaluate the effects of allelic series for breeding perspectives.

## Materials and Methods

### Plant Materials and Phenotyping

The association mapping panel consists of the Chinese peanut mini–mini core collection with 99 accessions and five additional accessions (Zhonghua 6_*vul*, Zhonghua 10_*vul*, Yuanza 9102_*vul*, ICG6375_*vul* and ICG12625_*aeq*) (Supplementary Table S1). These accessions included five botanic varieties namely var. *hypogaea*, var. *hirsuta*, var. *vulgaris*, var. *fastigiata*, and var. *aequatoriana*. These 104 accessions were planted in the experimental fields of Wuhan during three consecutive years (2012, 2013, and 2014) and during two consecutive years in Nanchong (2013 and 2014) in China using a randomly complete block design with two replications. Each accession was planted in a single-row with 12 plants within each row. The plant-to-plant spacing was 10 cm within each row and row-to-row spacing was 30 cm. Ten plants from each accession were randomly selected and surveyed for recording the observations for seed-related traits namely SL, SW, SL/W, and HSW according to previously described standard procedures (Jiang et al., 2006).

### Marker Polymorphism and Genotyping

Fresh leaves of each accession were collected for genomic DNA isolation. The quality detection of genomic DNA was performed using 1% agarose gel and uncut lambda DNA marker.

A total of 4,485 SSR markers obtained from the published literature (He et al., 2003; Ferguson et al., 2004; Moretzsohn et al., 2005; Cuc et al., 2008; Naito et al., 2008; Liang et al., 2009; Qin et al., 2012; Shirasawa et al., 2012, 2013; Tang et al., 2012; Wang et al., 2012; Zhang et al., 2012; Huang et al., 2016b; Zhou et al., 2016) were used to detect marker polymorphism in ten accessions selected based on their abundant phenotypic variation. Subsequently, the polymorphic markers segregating in a single-locus model were selected following the method previously described in *Brassica napus* (Xiao et al., 2012) and used to genotype the 104 accessions of the association panel. Polymerase chain reaction (PCR) amplifications, PCR products detection and estimation of fragment sizes were performed as described by Huang et al. (2016b). In some cases, in which it was difficult to distinguish polymorphic fragment because of size similarity, the PCR amplifications were re-conducted using SSR primers labeled with fluorescence dyes. The amplified PCR products were diluted to 10–50-folds based on the product concentration measured by agarose gel (1.2%), followed by mixing the diluted PCR products (1 μl) with GeneScan 500 LIZ standard (Applied Biosystems, 0.05 μl) with formamide (6.95 μl) in each well. Capillary electrophoresis (ABI 3730 Genetic Analyzer Applied Biosystems) was used to visualize the polymorphism. Output files were then transferred to computer and the allele sizing of the electrophoretic data was done using GeneMarker V2.1 software. The SSR allele was numerically coded in the ascending order ranked to the fragment size.

Heterozygous genotypes were treated as missing data. For each SSR, the rare alleles with minor allele frequency (MAF) ≤ 0.05 of which fragment sizes were apparently clustered were assigned into a common allelic class (Abdurakhmonov and Abdukarimov, 2008) and the other rare alleles which were unable to pool into common allelic class were treated as missing data to increase the power of association analysis (Hirschhorn and Daly, 2005). The markers with more than 10% missing data were excluded from the genetic analysis. The PIC of markers and gene diversity were calculated using the software PowerMarker 3.25 (Hirschhorn and Daly, 2005).

### Assessment of Population Structure and Relative Kinship

Population structure (*Q*-matrix) was estimated based on the polymorphic SSR markers using the software Structure 2.2 (Pritchard et al., 2000). It is a model-based clustering method for using multi-locus genotype data to infer population structure and assign individuals to groups. An admixture model with independent allele frequencies was applied to estimate each of the possible groups (*K*) from 1 to 10. To achieve reliable subpopulations, the other parameters were set at a higher level such as burn-in length of 1,00,000 followed by 1,00,000 iterations, with each *K* being run five times. In order to obtain the optimum *K*-value, a method calculating an *ad hoc* (Δ*K*) statistic based on the rate of change in LnP(D) between successive *K*-values was employed (Evanno et al., 2005). The accessions with membership probabilities ≥ 0.70 were assigned to corresponding group, otherwise they were classified into the mixed group. The Nei’s genetic distances were calculated to build unrooted neighbor-joining tree using PowerMarker 3.25 (Liu and Muse, 2005). A kinship coefficient estimation matrix was conducted using the SPAGeDi software package (Hardy and Vekemans, 2002).

### Linkage Disequilibrium

Generally, the correlation coefficient *r*^2^ was used to assess the LD using the software TASSEL version 3.0 (Bradbury et al., 2007). The significance of *r*^2^ was calculated based on Fisher’s exact test. Those SSRs mapped onto a high-density peanut genetic linkage map (Huang et al., 2016a) were selected and used to assess LD level in peanut. The pairs of markers located on the same linkage group were treated as linked markers, otherwise as unlinked markers. We calculated the LD levels for global, linked and unlinked markers, respectively. The background LD level for this population *per se* was defined as the 95th percentile of *r*^2^ distribution between all unlinked markers (Wu et al., 2016), which was regarded as a population-specific threshold to declare whether the LD is due to genetic linkage. The decay of LD with genetic distance was estimated by interval rather than marker-pairs individually for reducing the influence of outliers as previously described (Mather et al., 2007). We combined *r*^2^ values into an interval series of 0–0.5, 0.5–1, 1–2, 2–5, 5–10, 10–15, 15–20, 20–25, 25–50, 50–75, 75–100, and 100–150 cM based on marker distance. We estimated the averaged *r*^2^ for each interval and assumed the *r*^2^ value with 0-cM marker distance to be 1 as previously described (Yan et al., 2009). The non-linear regression function was deployed to fit the trend of LD decay. The LD decay was not only plotted in the whole genome, but also in the A and B subgenomes. The linked markers in the A and B subgenome were, respectively, selected and evaluated the LD levels using the method mentioned above in the whole genome.

### Statistical Analyses for Seed-Related Traits

Wuhan in 2012, Wuhan in 2013, Wuhan in 2014, Nanchong in 2013 and Nanchong in 2014 were treated as Environment I, II, III, IV, and V, respectively. The phenotypic distribution for seed-related traits, phenotypic correlations (*r*) between all traits and the effect of population structure on each trait were estimated using the R package^2^. Broad-sense heritability based on family mean was assessed as described by Jiang et al. (2014).

### Genome-Wide Association Study (GWAS) Analysis

The software program TASSEL3.0 (Bradbury et al., 2007) was used to conduct association analysis using a compressed mixed linear model (cMLM) by simultaneously accounting for population structure (*Q*-matrix) and relative kinship (*K*-matrix) to control the spurious associations (Zhang et al., 2010). In this study, we used an adjusted Bonferroni method to correct the multiple tests of association analysis, in which the *P*-value threshold was at *P* = 1.81 × 10^-3^ (1/*n, n* was the marker number for association analysis).

## Results

### Population Structure and Relative Kinship in the Peanut Panel

**FIGURE 1:**
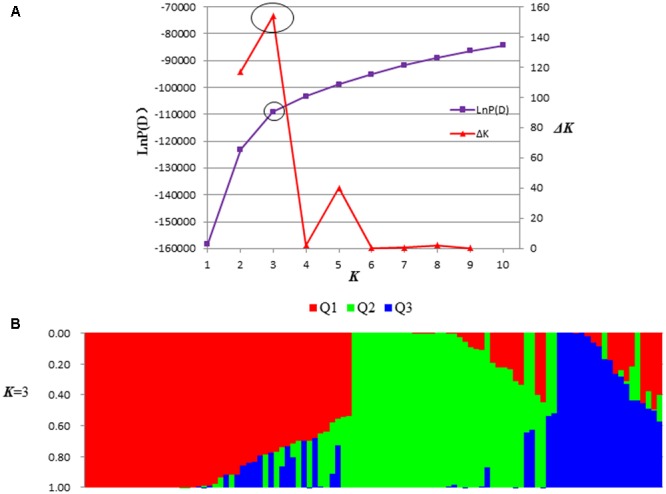
Population structure of the peanut panel. **(A)** Two different methods for determining the optimal value of *K*. The blue line indicated the *ad hoc* approach described by Pritchard et al. (2000). The red line indicated Δ*K* based on the change of LnP(D) between consecutive *K* and developed by Evanno et al. (2005). **(B)** The population structure in the peanut panel at *K* = 3.

**FIGURE 2:**
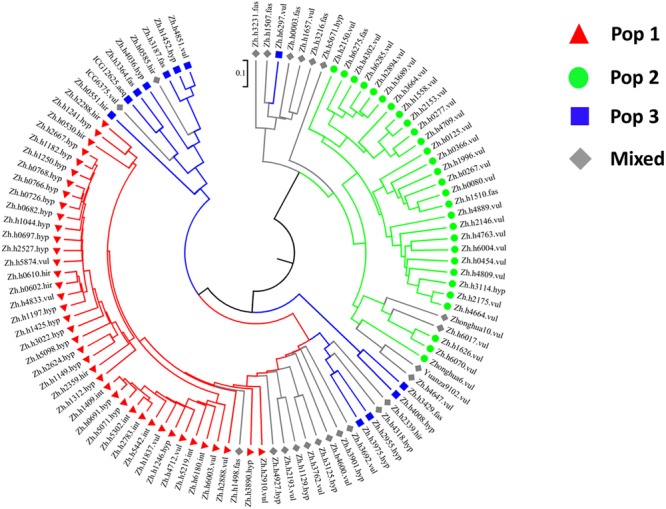
Dendrogram of the peanut panel based on SSR genotyping data.

Among 4,485 SSR markers, a total of 554 SSR markers were polymorphic segregated in a single-locus model in the peanut panel. Subsequently, these 554 polymorphic SSR markers were used to assess the population structure. The most significant change of the LnP(D) value was observed when *K* increased from 2 to 3, and a sharp peak of Δ*K* was observed at *K* = 3 (**Figure 1**). On the basis of the membership probabilities, all accessions were classified into any one of the three given groups or mixed group. The Pop 1 group contained 40 accessions (37.70%), in which 27 accessions (67.50%) belong to ssp. *hypogaea*. Twenty-nine accessions were classified into Pop 2 group, 96.60% of which belong to ssp. *fastigiata*. The third group, Pop 3, included 12 accessions, each half of which belongs to ssp. *hypogaea* and ssp. *fastigiata*, respectively. In the Mixed group, there were 8 (34.80%) and 15 (65.20%) accessions belonging to ssp. *hypogaea* and ssp. *fastigiata*, respectively. The clusters divided based on Nei’s genetic distances were basically consistent with the groups estimated by population structure analysis with a few exceptions (**Figure 2**).

**FIGURE 3:**
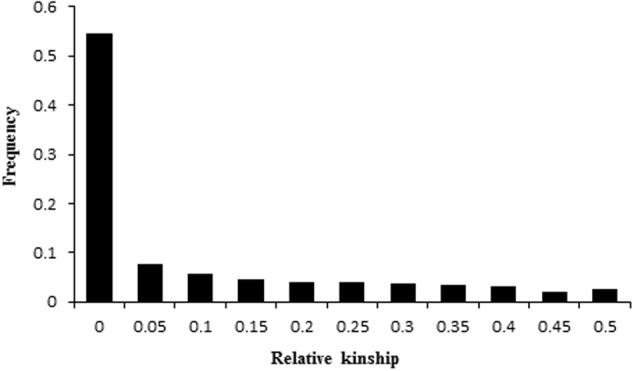
Distribution of pairwise relative kinship estimates. Only percentages of relative kinship estimates ranging from 0 to 0.5 were shown.

Relative kinship within the population was evaluated based on 554 single-locus SSR markers. The average relative kinship value in the peanut panel was 0.11. More than 70% of the pairwise kinship estimates were below 0.05, with a continuously declining frequency of kinship values falling in higher kinship assessment categories (**Figure 3**), indicating that there was no or very weak genetic relationship among these accessions.

### Linkage Disequilibrium Decay in Peanut Genome

The extent of LD in the peanut association panel was assessed using *r*^2^ of 274 SSR loci mapped onto 20 linkage groups (Huang et al., 2016a). In total panel, the average *r*^2^ was 0.10 and almost 67.10% of *r*^2^ values showed statistically significant (*P* < 0.001) for global markers. Moreover, we found that a higher level of LD (i.e., the significant *r*^2^ proportion and average *r*^2^ value) was found between the linked markers than that between unlinked markers in each group and the whole panel (**Table 1**), respectively, which confirmed that genetic linkage strongly shaped LD rather than random effects.

**Table 1:** Linkage disequilibrium (LD) in the entire panel and the four subpopulations.

Population^a^	*N*^b^	Global^c^		Unlinked^d^		Linked^e^	
		*r*^2^	Significant LD (%)^f^	*r*^2^	Significant LD (%)^f^	*r*^2^	Significant LD (%)^f^
Pop 1	40	0.05	2.44	0.05	1.82	0.14	14.24
Pop 2	29	0.07	2.95	0.06	2.31	0.13	14.22
Pop 3	12	0.17	0.50	0.17	0.47	0.19	1.10
Mixed	23	0.08	1.87	0.07	1.31	0.13	12.13
Total	104	0.10	67.10	0.10	66.47	0.16	78.14

*^*a*^The overall 104 peanut accessions were classified into Pop 1, Pop 2, Pop 3 and Mixed using the structural analysis. ^*b*^The number of accessions for overall and each inferred groups. ^*c*^The whole set of marker pairs, including linked and unlinked markers pairs. ^*d*^Pairs of markers from different linkage groups. ^*e*^Pairs of markers on the same linkage groups. ^*f*^Significant threshold is set to *P* < 0.001, which determine whether pairwise LD estimate is significant statistically.*

**FIGURE 4:**
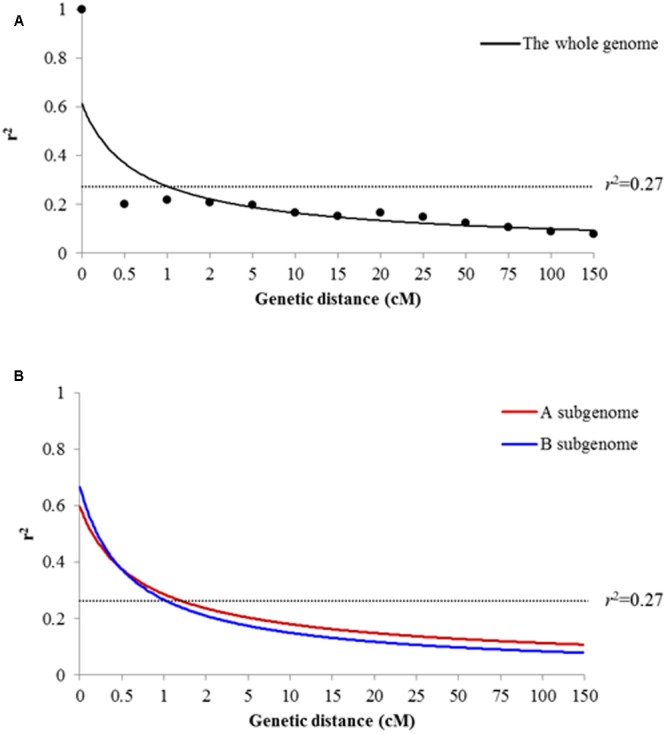
Linkage disequilibrium (LD) decay for all the 104 accessions. **(A)** Genome-wide LD decay of the whole genome for the peanut panel. **(B)** LD decay of the A and B subgenomes for the peanut panel.

To obtain a population-specific *r*^2^ cutoff for LD decay, we collected all *r*^2^ values between unlinked marker-pairs and estimated the 95th percentile of which distribution as the background LD level, i.e., *r*^2^ = 0.27, in preset study. Thus, we found that the whole-genome LD decayed to 1 cM at background level of *r*^2^ = 0.27 in present peanut association panel (**Figure 4A**). LD of B subgenome decayed faster than that of A subgenome (**Figure 4B**).

### Genetic Diversity in the Peanut Panel

A total of 554 SSR markers (Supplementary Table S2) were polymorphic and segregated in a single-locus model in the association panel. Eventually, 1,950 alleles were obtained in the population ranging from 2 to 12 with an average of 3.50 alleles per locus (Supplementary Table S2). The average PIC of the SSR markers in the whole peanut panel was 0.47 ranging from 0.10 (AHGS2445) to 0.90 (AHGS1163). Although the Pop 1 group contained the most accessions, it had the lowest level of alleles per locus (2.70), gene diversity (0.25), and PIC (0.22). In the four subgroups, the Mixed group had the lowest major allele frequency (0.58) and the highest level of alleles per locus (3.40), gene diversity (0.53), and PIC (0.47) (**Table 2**).

**Table 2:** Diversity-related summary statistics for all groups defined by structural analysis.

Population^a^	*N*^b^	Alleles number of	Alleles number of	Major allele	Genetic	PIC
		all markers	each marker	frequency	diversity	
Total^a^	104	1950	3.50	0.57	0.54	0.47
Pop 1	40	1480	2.70	0.84	0.25	0.22
Pop 2	29	1502	2.70	0.75	0.34	0.30
Pop 3	12	1571	2.80	0.61	0.48	0.42
Mixed	23	1862	3.40	0.58	0.53	0.47

*^*a*^The overall 104 peanut accessions were classified into Pop 1, Pop 2, Pop 3 and Mixed using the structural analysis. ^*b*^The number of accessions for overall and each inferred groups.*

### Phenotypic Variation of Seed-Related Traits

**FIGURE 5:**
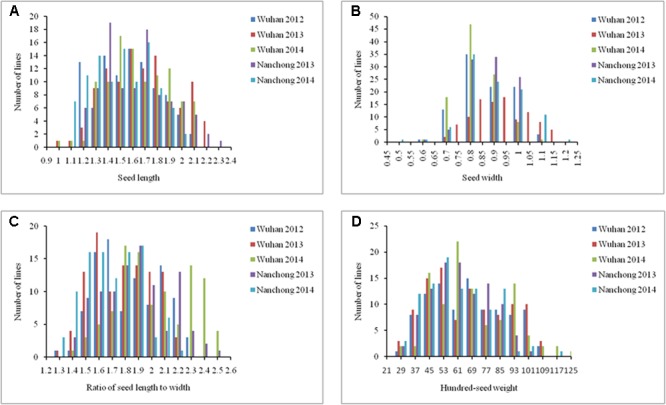
Phenotypic distribution of 4 seed-related traits in the peanut panel in the five different environments. **(A)** Seed length; **(B)** Seed width; **(C)** Ratio of seed length to width; **(D)** Hundred-seed weight.

Large variation was observed among the 104 accessions in five different environments for SL, SW, ratio of SL to width and HSW (**Figure 5**). Coefficient of variation (CV) for HSW was higher than other three seed-related traits in five different environments. The highest CV (35.60%) was observed in HSW in environment II, which varied from 21.50 to 108.00 g with an average of 63.03 g. The lowest CV (10.55%) was found in SW in environment I, which varied from 0.70 to 1.00 cm with an average of 0.85 cm (**Table 3**).

**Table 3:** Phenotypic variation for the four seed-related traits in the peanut panel.

Trait^a^	Environment^b^	Minimum	Maximum	Mean	*SD*^c^	CV(%)^d^	*R*^2^(%)^e^	*H*^2f^
SL	I	1.20	2.10	1.57	0.25	15.78	54.69	0.94
	II	0.98	2.18	1.62	0.29	17.60	57.22	
	III	1.00	2.10	1.65	0.25	15.39	57.69	
	IV	1.20	2.30	1.63	0.27	16.75	51.70	
	V	1.10	2.00	1.50	0.24	16.11	55.06	
SW	I	0.60	1.10	0.86	0.11	12.86	30.21	0.85
	II	0.67	1.13	0.91	0.11	12.28	41.04	
	III	0.60	1.10	0.83	0.09	11.01	13.70	
	IV	0.60	1.00	0.88	0.09	10.55	31.46	
	V	0.50	1.20	0.89	0.13	14.24	33.93	
SL/W	I	1.30	2.30	1.84	0.24	13.20	46.00	0.78
	II	1.30	2.20	1.78	0.22	12.58	49.63	
	III	1.40	2.50	2.02	0.28	13.80	51.01	
	IV	1.33	2.56	1.86	0.26	14.06	38.20	
	V	1.30	2.20	1.70	0.21	12.32	41.40	
HSW	I	24.50	103.00	63.46	20.56	32.40	47.83	0.93
	II	21.50	108.00	63.03	22.44	35.60	51.64	
	III	25.60	117.80	64.97	20.80	32.02	42.32	
	IV	25.80	95.90	58.12	16.67	28.68	46.99	
	V	21.30	114.50	56.99	18.13	31.81	41.53	

*^*a*^SL, seed length; SW, seed width; SL/W, ratio of seed length to width; HSW, hundred-seed weight. ^*b*^I: Wuhan in 2012; II: Wuhan in 2013; III: Wuhan in 2014; IV: Nanchong in 2013; V: Nanchong in 2014. ^*c*^Standard deviation. ^*d*^Coefficients of variation. ^*e*^Percentage of phenotypic variance explained by population structure. ^*f*^Broad-sense heritability of each trait.*

The broad-sense heritability of the four seed-related traits was 0.94, 0.85, 0.78, and 0.93 for SL, SW, SL/W, and HSW, respectively (**Table 3**), which was relatively high indicating that genetic factors played a predominant role in determining the variation for these traits. In addition, population structure had significant effect on the seed-related traits, explaining phenotypic variation from 13.70% for SW in environment III to 57.69% for SL in environment III (**Table 3**). Significantly positive correlations were discovered in all pairs of the four seed-related traits except SW didn’t correlated to SL/W (**Table 4**). The SL, SW, and SL/W strongly correlated with HSW (*r* = 0.88, 0.90, and 0.33, respectively, *P* < 0.01), which indicated the HSW is directly influenced by seed size. The positive correlation between SL and SW (*r* = 0.66, *P* < 0.01) implied that these traits may be affected by the same genetic factors. SL/W strongly correlated with SL (*r* = 0.71, *P* < 0.01) but not with SW (*r* = -0.04), which indicated that the seed shape was dominated by SL.

**Table 4:** Correlation analysis for the seed-related traits.

	SL	SW	SL/W	HSW
SL		0.66	0.71	0.88
SW	^∗∗^		-0.04	0.90
SL/W	^∗∗^	ns		0.33
HSW	^∗∗^	^∗∗^	^∗∗^	

*SL, seed length; SW, seed width; SL/W, ratio of seed length to width; HSW, hundred-seed weight. ^∗∗^Significant at *P* < 0.01; ns, non-significant at *P* < 0.05.*

### Genome-Wide Association Mapping for Four Seed-Related Traits

A total of 30 MTAs were identified for the four seed-related traits in five environments at significant level of *P* < 1.81 × 10^-3^ (**Table 5** and **Supplementary Figure S1**) with phenotypic variance explained (PVE) ranging from 11.22 to 32.30%. Nine markers significantly associated with SL were detected in five environments with PVE ranging from 11.64 to 31.45%. Among these associated markers, AGGS1312 and AHGA44686 were repeatedly detected in three and two environments, respectively. AHGA44686 detected in Environment IV had the largest effect on SL (PVE = 31.45%), with the largest phenotypic difference between alleles of 0.62 cm, almost equivalent to one-half of the minimum SL collected in peanut diverse panel. For SW, only two markers, AHGS2155 and AHGS1836, were identified only in single environment with PVE of 17.48 and 19.60%, respectively. There were ten markers significantly associated with SL/W with PVE ranging from 11.22 to 22.02%, among which two markers associated with SL/W (AGGS1272 and AHGS2191) were repeatedly detected in two environments. AGGS1272 detected in Environment V had the largest effect (PVE = 22.02%) with the largest phenotypic difference between alleles of 1.07. Nine markers were significantly associated with HSW in any of given five environments with PVE ranging from 11.61 to 32.30%. Among which, AHGA44686 was repeatedly identified in four environments with PVE ranging from 26.46 to 32.30%. AHGA44686 detected in environment I had the largest effect (PVE = 32.30%) with the largest HSW between alleles difference of 36.44 g. In brief, five markers associated with one of the four seed-related traits were repeatedly detected in at least two environments. In addition, two markers (AHGA44686 and AGGS2359) were found commonly associated with multiple seed-related traits, probably reflecting the positive correlations among these traits. It was notable that AHGA44686 was simultaneously and repeatedly associated with SL and HSW in multiple environments with large explained phenotypic variance (26.23 ∼ 32.30%).

**Table 5:** Marker-trait associations detected with four seed-related traits (*P* < 1.81 × 10^-3^).

Trait^a^	Marker^b^	Genetic position^c^		Environment^d^	*P*-value	PVE(%)^e^
		LG.	Pos. (cM)			
SL	**AGGS1312**	B10	19.21	II	1.42E-03	13.32
	**AGGS1312**	B10	19.21	III	1.21E-04	19.41
	**AGGS1312**	B10	19.21	V	2.37E-04	17.34
	AGGS1717	B01	71.44	IV	3.03E-04	20.69
	AGGS2359	B01	73.60	IV	7.77E-04	11.89
	AHGS2466	B01	74.55	IV	7.69E-04	11.64
	**AHGA44686**			II	6.73E-04	26.23
	**AHGA44686**			IV	2.44E-04	31.45
	EM88	B04	56.37	II	2.50E-04	12.82
SW	AHGS2155			IV	2.46E-04	17.48
	AHGS1836			II	1.25E-03	19.60
SL/W	AGGS1126	A04	37.50	IV	1.10E-03	11.22
	**AGGS1272**			I	1.38E-03	18.84
	**AGGS1272**			V	7.08E-04	22.02
	AGGS2359	B01	73.60	IV	1.39E-03	11.29
	AGGS2390	B08	29.81	IV	1.42E-03	19.23
	AHGS1625	B01	74.02	IV	1.45E-03	14.12
	AHGS1650			IV	3.40E-04	21.54
	**AHGS2191**	B10	18.38	I	9.25E-04	12.77
	**AHGS2191**	B10	18.38	IV	1.31E-03	11.66
	ARS590			IV	1.45E-03	13.98
HSW	4F7			III	7.26E-04	11.61
	AGGS1802	B10	11.90	I	3.15E-04	18.95
	AHGS2561			II	1.69E-03	12.58
	AHGS2787			I	8.64E-04	18.78
	**AHGA44686**			I	5.26E-04	32.30
	**AHGA44686**			II	2.93E-04	29.27
	**AHGA44686**			IV	4.80E-04	28.74
	**AHGA44686**			V	1.47E-03	26.46
	GM1854			V	2.09E-04	17.96

*^*a*^SL, seed length; SW, seed width; SL/W, ratio of seed length to width; HSW, hundred-seed weight. ^*b*^Bolds are associated markers detected in at least two environments. ^*c*^Genetic position of markers on the genetic linkage map constructed by Huang et al. (2016a). ^*d*^I: Wuhan in 2012; II: Wuhan in 2013; III: Wuhan in 2014; IV: Nanchong in 2013; V: Nanchong in 2014. ^*e*^The phenotypic variation explained by each significant marker.*

### The Accumulation of Favorable Alleles

For seed-related traits, the favorable alleles for breeder are defined as the ones conferring high phenotypic values in peanut. Overall, there were 9 favorable alleles for SL, 2 favorable alleles for SW, 10 favorable alleles for SL/W and 9 favorable alleles for HSW (Supplementary Table S3). Among these favorable alleles, the favorable allele AHGA44686-259 detected in Environment II (*P* = 6.73 × 10^-4^, PVE = 26.23%) and Environment IV (*P* = 2.44 × 10^-4^, PVE = 31.45%) which was present in 18 accessions enabled the SL to become the highest (1.92 cm) in both environments. A total of 47 accessions possessing the favorable allele AHGS1836-274 detected in environment II (*P* = 1.25 × 10^-3^, PVE = 19.60%) had the highest SW (0.97 cm). Eight accessions possessing the favorable allele AGGS1126-202 detected in environment IV (*P* = 1.10 × 10^-3^, PVE = 11.22%) had the highest SL/W (2.18) and 6 accessions possessing the favorable allele GM1854-115 detected in environment V had the highest HSW (88.76 g).

The 104 accessions were partitioned based on haplotypes built by two associated markers to assess their combined effects on seed-related traits (Supplementary Table S4). AGGS1312 (X locus) and AHGA44686 (Y locus) were associated with SL in Environment II and they detected 3 (X01, X02 and X03) and 8 (Y01, Y02, Y03, Y04, Y05, Y06, Y07, and Y08) alleles, respectively. We totally obtained 24 haplotypes between AGGS1312 and AHGA44686 across 104 accessions, only leaving 7 haplotypes (*n* > 5) for effect estimation by multiple comparison analysis. X03Y04 had the highest SL (1.94 ± 0.17 cm, *n* = 14), followed by X03Y03 (1.85 ± 0.31 cm, *n* = 14), X03Y02 (1.74 ± 0.10 cm, *n* = 6), X03Y01 (1.69 ± 0.09 cm, *n* = 6), X01Y06 (1.56 ± 0.12 cm, *n* = 5), X01Y07 (1.50 ± 0.19 cm, *n* = 12), X01Y08 (1.37 ± 0.14 cm, *n* = 8). For SL/W, the alleles at AGGS1272 (X locus) and AHGS2191 (Y locus) formed eight combined genotypes in Environment I, X1Y1, X1Y2, X2Y1, X2Y2, X3Y1, X3Y2, X4Y1, and X4Y2, among which, X1Y2, X3Y1, X3Y2, and X4Y2 were eliminated because of the little accessions (*n* < 5). X4Y1 had the highest SL/W (2.18 ± 0.10, *n* = 5), followed by X1Y1 (2.10 ± 0.10, *n* = 23), X2Y1 (1.79 ± 0.20, *n* = 18), and X2Y2 (1.64 ± 0.10, *n* = 28).

## Discussion

### The Usefulness of Peanut Mini–Mini Core Collection for Diversity and Association Mapping

Previously, we developed a peanut mini-core collection of 298 accessions that were selected from more than 6,000 accessions deposited in genebank of OCRI-CAAS (Jiang et al., 2014). In the present study, we typically used a smaller panel that included a mini–mini core set of 99 accessions (Jiang et al., 2013) and 5 exotically diverse accessions in a comprehensive genetic analysis. The experimental design is based on several considerations as follows: (1) we want to genotype more markers to better tag LD relations between markers and underlying genes; (2) we expect to phenotype the quantitative traits in more environments to better control the environmental bias. So, given funding and technical limitations, we successfully genotype largely more markers (nearly sixfold) and collect phenotype in more environments (5 vs. 3) compared to the previous study based mini-core collection of 298 accessions. Given the higher genetic diversity of 104 than 298 accessions (0.54 vs. 0.265) (**Table 2**; Jiang et al., 2014), we expect this delicately assembled panel may be useful to increase the mapping power because it enables to significantly increase marker density and calibrate environmental errors. However, we understand that some QTLs with minor effects may be missed due to sample size decrease. The advance of technologies in genotyping and phenotyping will allow us to thoroughly dissect the genetics of quantitative traits in larger populations.

In the present study, the assembled peanut panel of 104 accessions totally detected 1,950 alleles using 554 single-locus SSR markers, with an average of 3.50 alleles per locus. We found the number of alleles per locus is slightly lower than that in the peanut mini core collection of 298 accessions (Jiang et al., 2014), and significantly lower than that identified in United States peanut mini core collection (Wang et al., 2011) and ‘Reference Set’ comprising 300 genotypes (Pandey et al., 2014). The genetic drift should be one source for this difference as the smaller population basically has a rare opportunity to sample more allelic types. The number and type of SSR may be another potential source, because the preference of single-locus SSR in current study may underestimate the value after significantly reducing the genotyping complexity (Vigouroux et al., 2002). The gene diversity of the peanut panel in this study (0.54) was similar to that of United States peanut mini core collection (Wang et al., 2011), but significantly lower than ‘Reference Set’ (Pandey et al., 2014), which may be caused by the difference between their germplasm backgrounds (Fukunaga et al., 2005). Interestingly, we found the present panel showed an apparently higher gene diversity than the peanut mini core collection with a highly similar genetic background (Jiang et al., 2014). We expect that the assembly of the current panel may reform the allelic spectrums, largely reducing low-frequency alleles in 104 accessions compared to 298 accessions, which would globally elevate the diversity estimates per locus.

### LD Decay in the Peanut Panel

In peanut, LD was roughly estimated to decay within 10 cM using 32 SSR primers (Belamkar et al., 2011). In the present study, we updated the decay distance of LD to be 1 cM or 1.3 Mb using 274 single-locus SSR loci in peanut. Comparatively, LD decayed apparently slower in peanut than maize (5 Kb) (Yan et al., 2009), this should be driven by differing mating systems; while it was slightly slower than rice (200 Kb) (Huang et al., 2010) perhaps due to lack of sufficient markers to totally cover the peanut genome. However, we found the decay distances were comparable between peanut and rapeseed (1.2 Mb) (Wu et al., 2016) although they were mated in different ways. This may be attributed to the relatively narrow genetic background in that rapeseed population, which could be seen that the estimated background LD extent in rapeseed was as high as in peanut (i.e., *r*^2^ = 0.26 and *r*^2^ = 0.27). The LD of B subgenome decayed faster than that of the A subgenome, which gave the clues that historical recombination events may more frequently happen in B subgenome rather than A subgenome. As shown in LD heatmaps (**Supplementary Figure S2**), we could not observed any apparent LD block on the neither chromosome, which may be caused by the low LD between pairwise markers. To clearly depict the haplotype blocks, marker density in the present study is still insufficient.

It needs to note that, the limited population size (i.e., 104 lines) would be hard to avoid the statistical bias in estimating LD in present study, because of bottleneck effect and genetic drift (Flint-Garcia et al., 2003). The high LD and slowly LD decay maybe attributed to the small population used in this study, which will cause the low resolution of association analysis. As faster LD decay imply the higher mapping resolution, thus we could expect that the sufficient resolution may be achieved in even self-pollinated species if the mapping population was carefully assembled that specially included diverse genetic backgrounds.

### Marker-Trait Associations (MTAs) for Seed-Related Traits in Peanut

Seed weight is controlled by a combination seed features such as SL, SW, and seed thickness. Several genes for seed-related traits have been obtained in many crops using the forward genetic strategy and reverse genetic strategies (Li et al., 2011; Liu et al., 2015; Si et al., 2016). In the previous studies, several QTLs were identified in peanut using SSR markers and *F*_2:3_ populations for SL, SW, and seed weight (Shirasawa et al., 2012; Huang L. et al., 2015; Chen et al., 2016). However, the utilization of *F*_2:3_ populations made it impossible to identify consistent and stable QTLs across locations and seasons for seed-related traits and decreased the resolution of linkage mapping due to lack of multiple environments. Therefore, in this study, we performed association mapping for seed-related traits in multiple environments containing multiple locations and seasons. Eventually, a total of 30 MTAs for seed-related traits were detected with PVE ranging from 11.22 to 32.30% using cMLM model which was sufficient to minimize false-positive associations. Of these, 5 markers were consistently identified in 2, 3, or 4 environments (*P* < 1.81 × 10^-3^, **Table 5**), indicating that the QTLs associated with these markers were insensitive to the multi-environments. In the “Reference Set” comprising 300 genotypes, 9, 3, and 5 markers were detected for SL, SW, and seed weight, respectively (Pandey et al., 2014). In Chinese peanut mini core collection, 4, 2, and 8 markers were associated with SL, SW, and HSW, respectively (Jiang et al., 2014). The markers associated with seed-related traits in our study were inconsistent with those ones reported in the previous studies on linkage mapping and association mapping and were considered as novel markers identified for seed-related traits. The differences of markers associated with seed-related traits were caused by the different germplasms and the different SSR markers used in previous and this study. In addition, the marker AHGA44686 was simultaneously and repeatedly associated with SL and HSW in multiple environments with large PVE (26.23 ∼ 32.30%), indicating that AHGA44686 is promising genetic marker which can increase HSW via SL.

The combination of alleles from a few significant loci had the potential of explaining much larger phenotypic variation (Jia et al., 2012; Cai et al., 2014). In our study, AGGS1312 and AHGA44686 explained 13.32 and 26.23% of phenotypic variation of SL in environment II, respectively, while they jointly explained 62.00% of phenotypic variation for SL. This indicated that the seed-related traits may be inherited in an additive manner (He et al., 2010; Cai et al., 2014), which made the breeding for these traits more complicated. Additionally, the accession (Zh.h6004_*vul*) possessing seven favorable alleles of SL/W associated loci had the highest SL/W (2.56), while the accession (Zh.h6275_*fas*) possessing no favorable allele of SL/W associated loci had the lowest SL/W (1.33) in environment IV. For HSW, the accession (Zh.h5302_*int*) possessing two favorable alleles of associated loci had the highest HSW (114.50 g), while three accessions (Zh.h3216_*fas*, Zh.h1312_*hyp*, and Zh.h0551_*hir*) containing no favorable alleles had the lowest HSW (21.30, 23.00, and 29.00 g, respectively) in environment V. These results suggested that the accumulation of favorable alleles could effectively enhance trait performance of peanut variety for seed-related traits and applicate in peanut yield molecular breeding.

## Conclusion

In this study, a highly diverse peanut panel consisting of 104 accessions was used to perform association mapping for seed-related traits. It was firstly reported that the LD of B subgenome decayed faster than that of the A subgenome. Large phenotypic variation was observed for four seed-related traits in the association panel. Using mixed linear model with population structure and kinship, a total of 30 significant single-locus SSR markers were detected to be associated with four seed-related traits (*P* < 1.81 × 10^-3^) across five different environments. The favorable alleles of associated markers for each seed-related trait and the optimal combination of favorable alleles of associated markers were identified to significantly enhance trait performance, revealing a potential of utilization of these associated markers in peanut breeding program.

## Author Contributions

HJ and BL designed the experiment. XR and YX planted the mapping population. BW performed SSR polymorphism detection. JZ and ZL performed SSR genotyping in the mapping population. LH, XR, YC, XZ, WC, and YX performed seed-related traits evaluation in the mapping population. JZ and LH assessed the population structure, relative kinship and linkage disequilibrium level in the peanut panel, and performed association analysis. JZ, LH, and MP wrote the manuscript. HL, YL, RV, BL, and HJ helped in interpreting the results and revised the manuscript. All of the authors read and approved the final manuscript.

## Conflict of Interest Statement

The authors declare that the research was conducted in the absence of any commercial or financial relationships that could be construed as a potential conflict of interest.

## Footnotes

1 http://faostat.fao.org/

2 http://www.R-project.org/

## Supplementary Material

FIGURE S1 Manhattan plots of association analysis using the mixed linear model (MLM) for four traits. **(A)** Seed length (SL); **(B)** seed width (SW); **(C)** ratio of seed length to width (SL/W); and **(D)** hundred-seed weight (HSW). The blue plots, red plots, green plots, purple plots, light blue plots represent the associated signals for five different environments (Wuhan in 2012, Wuhan in 2013, Wuhan in 2014, Nanchong in 2013, and Nanchong in 2014), respectively. The black dashed horizontal line depict the significant thresholds that is -log_10_1/554 = 2.74.

FIGURE S2 Linkage disequilibrium (LD) heatmap in 20 linkage groups. The markers in red are the significant association signals.
