# Supplementary figures and images for: Genetic Variation and Association Mapping of Seed-Related Traits in Cultivated Peanut (Arachis hypogaea L.) Using Single-Locus Simple Sequence Repeat Markers
Source: Front Plant Sci. 2017 Dec 11;8:2105. doi: 10.3389/fpls.2017.02105 (PMC5732145; doi:10.3389/fpls.2017.02105)

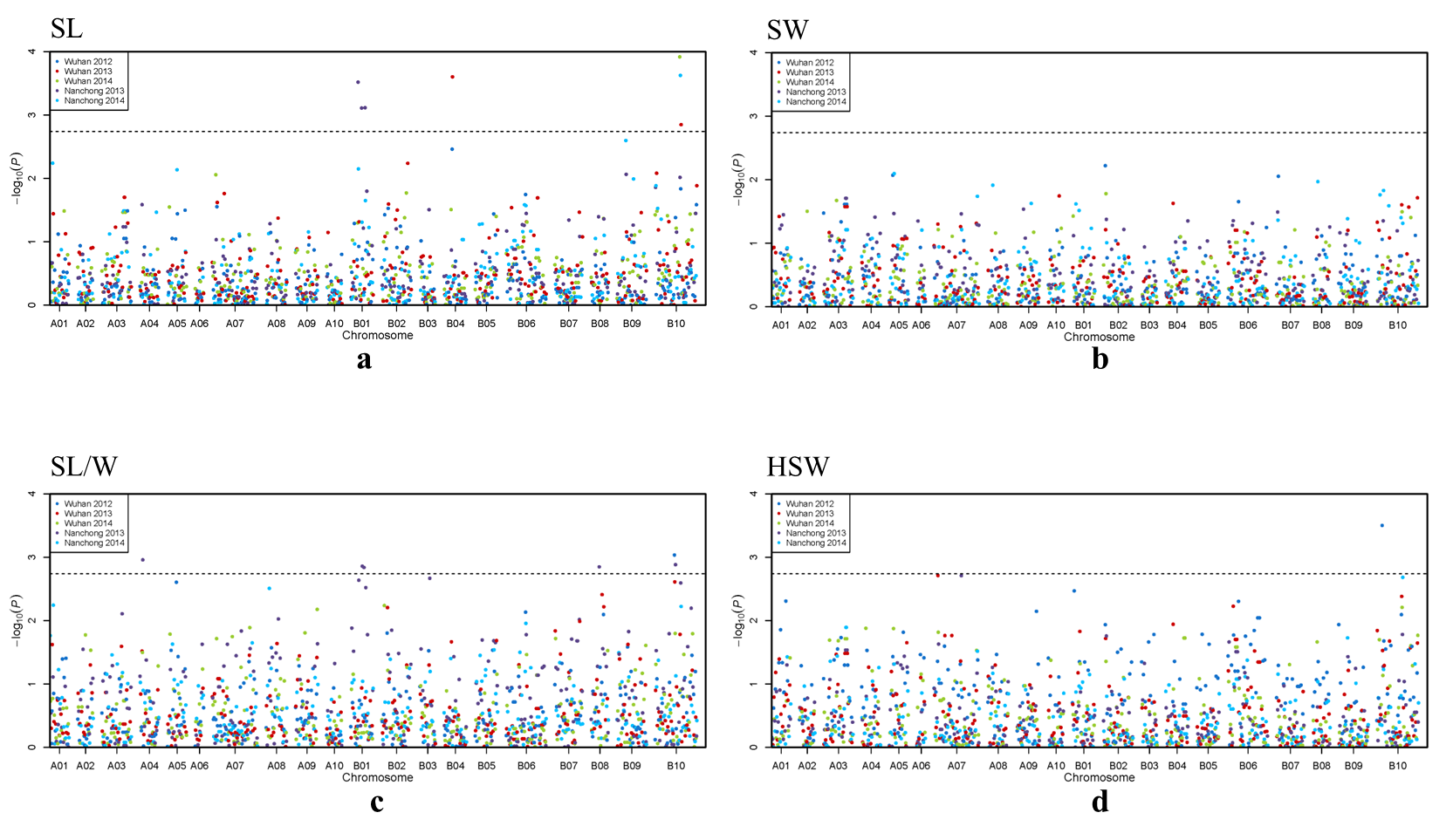

Supplement: FIGURE S1 — Manhattan plots of association analysis using the mixed linear model (MLM) for four traits. (A) Seed length (SL); (B) seed width (SW); (C) ratio of seed length to width (SL/W); and (D) hundred-seed weight (HSW). The blue plots, red plots, green plots, purple plots, light blue plots represent the associated signals for five different environments (Wuhan in 2012, Wuhan in 2013, Wuhan in 2014, Nanchong in 2013, and Nanchong in 2014), respectively. The black dashed horizontal line depict the significant thresholds that is -log101/554 = 2.74. [file Image_1.TIF]
