# Supplementary material for: Genetic Variation and Association Mapping of Seed-Related Traits in Cultivated Peanut (Arachis hypogaea L.) Using Single-Locus Simple Sequence Repeat Markers
Source: Front Plant Sci. 2017 Dec 11;8:2105. doi: 10.3389/fpls.2017.02105 (PMC5732145; doi:10.3389/fpls.2017.02105)
Supplement: FIGURE S2 — Linkage disequilibrium (LD) heatmap in 20 linkage groups. The markers in red are the significant association signals. [file Image_2.PDF]

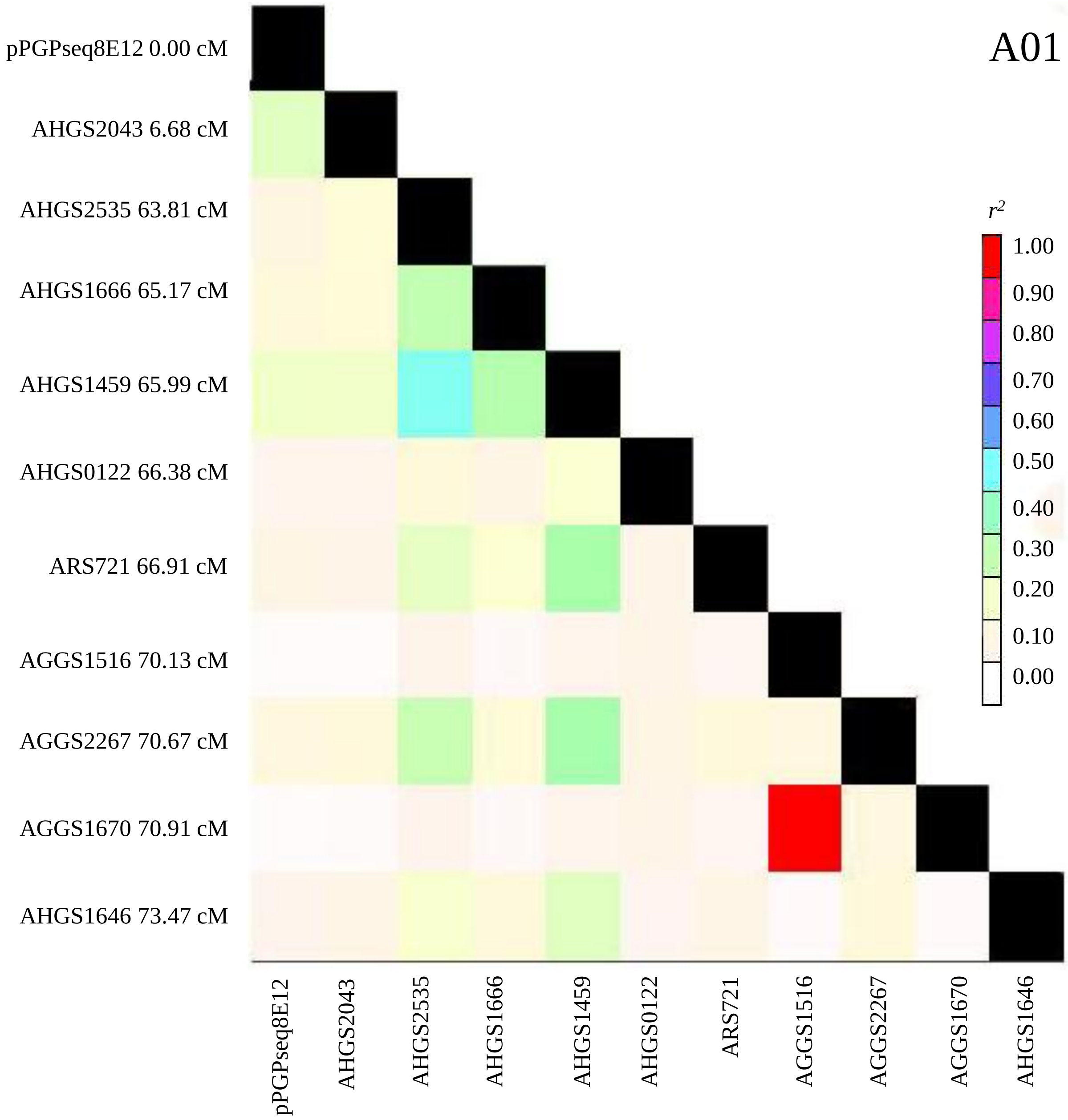
A01
pPGPseq8E12 0.00 cM
AHGS2043 6.68 cM
AHGS2535 63.81 cM
AHGS1666 65.17 cM
AHGS1459 65.99 cM
AHGS0122 66.38 cM
ARS721 66.91 cM
AGGS1516 70.13 cM
AGGS2267 70.67 cM
AGGS1670 70.91 cM
AHGS1646 73.47 cM
pPGPseq8E12
AHGS2043
AHGS2535
AHGS1666
AHGS1459
AHGS0122
ARS721
AGGS1516
AGGS2267
AGGS1670
AHGS1646
r^2
1.00
0.90
0.80
0.70
0.60
0.50
0.40
0.30
0.20
0.10
0.00

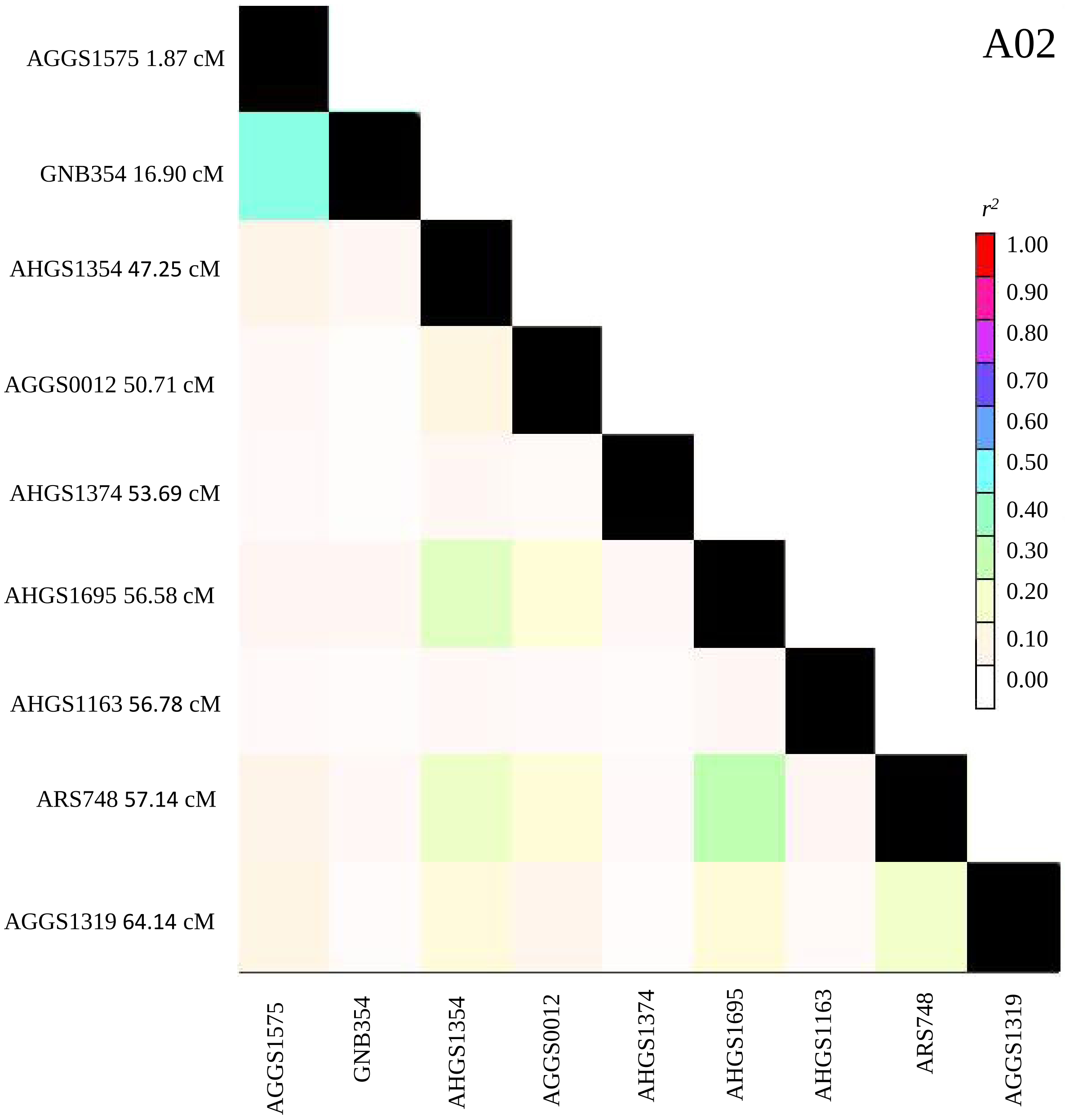
A02
AGGS1575 1.87 cM
GNB354 16.90 cM
AHGS1354 47.25 cM
AGGS0012 50.71 cM
AHGS1374 53.69 cM
AHGS1695 56.58 cM
AHGS1163 56.78 cM
ARS748 57.14 cM
AGGS1319 64.14 cM
r2
1.00
0.90
0.80
0.70
0.60
0.50
0.40
0.30
0.20
0.10
0.00
AGGS1575
GNB354
AHGS1354
AGGS0012
AHGS1374
AHGS1695
AHGS1163
ARS748
AGGS1319

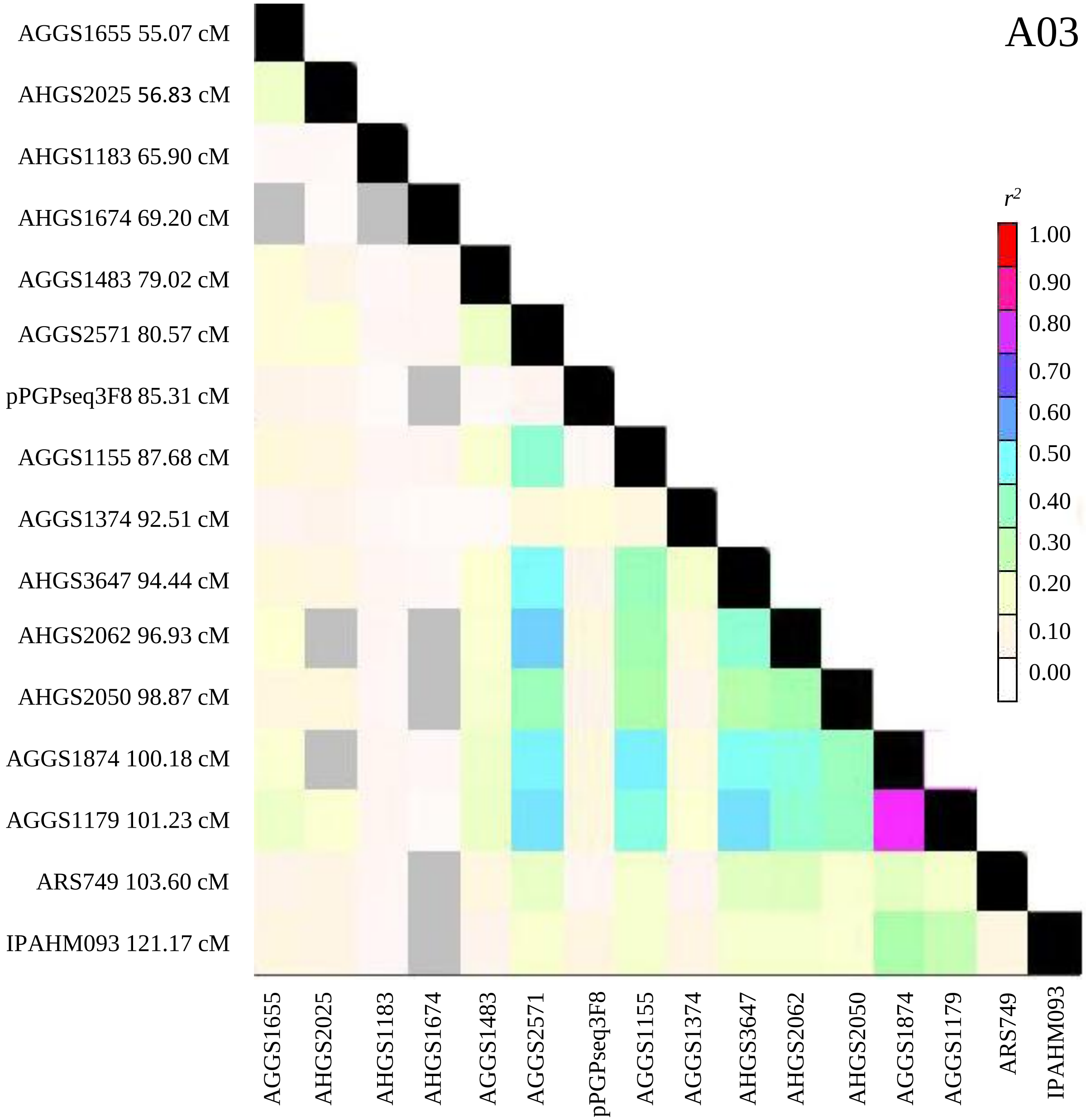
A03
AGGS1655 55.07 cM
AHGS2025 56.83 cM
AHGS1183 65.90 cM
AHGS1674 69.20 cM
AGGS1483 79.02 cM
AGGS2571 80.57 cM
pPGPseq3F8 85.31 cM
AGGS1155 87.68 cM
AGGS1374 92.51 cM
AHGS3647 94.44 cM
AHGS2062 96.93 cM
AHGS2050 98.87 cM
AGGS1874 100.18 cM
AGGS1179 101.23 cM
ARS749 103.60 cM
IPAHM093 121.17 cM
AGGS1655
AHGS2025
AHGS1183
AHGS1674
AGGS1483
AGGS2571
pPGPseq3F8
AGGS1155
AGGS1374
AHGS3647
AHGS2062
AHGS2050
AGGS1874
AGGS1179
ARS749
IPAHM093
r^2
1.00
0.90
0.80
0.70
0.60
0.50
0.40
0.30
0.20
0.10
0.00

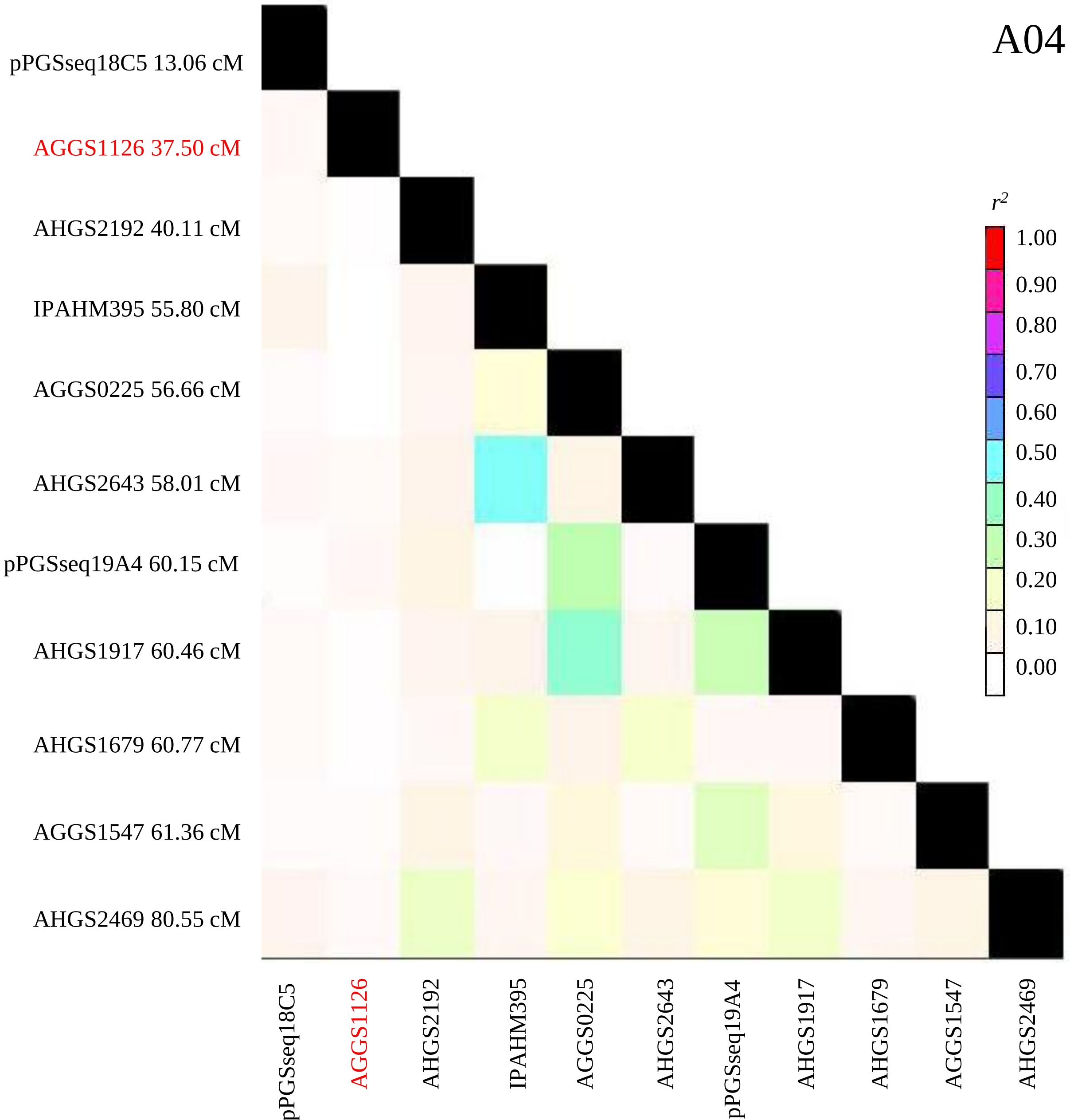
A04
pPGSseq18C5 13.06 cM
AGGS1126 37.50 cM
AHGS2192 40.11 cM
IPAHM395 55.80 cM
AGGS0225 56.66 cM
AHGS2643 58.01 cM
pPGSseq19A4 60.15 cM
AHGS1917 60.46 cM
AHGS1679 60.77 cM
AGGS1547 61.36 cM
AHGS2469 80.55 cM
pPGSseq18C5
AGGS1126
AHGS2192
IPAHM395
AGGS0225
AHGS2643
pPGSseq19A4
AHGS1917
AHGS1679
AGGS1547
AHGS2469
r^2
1.00
0.90
0.80
0.70
0.60
0.50
0.40
0.30
0.20
0.10
0.00

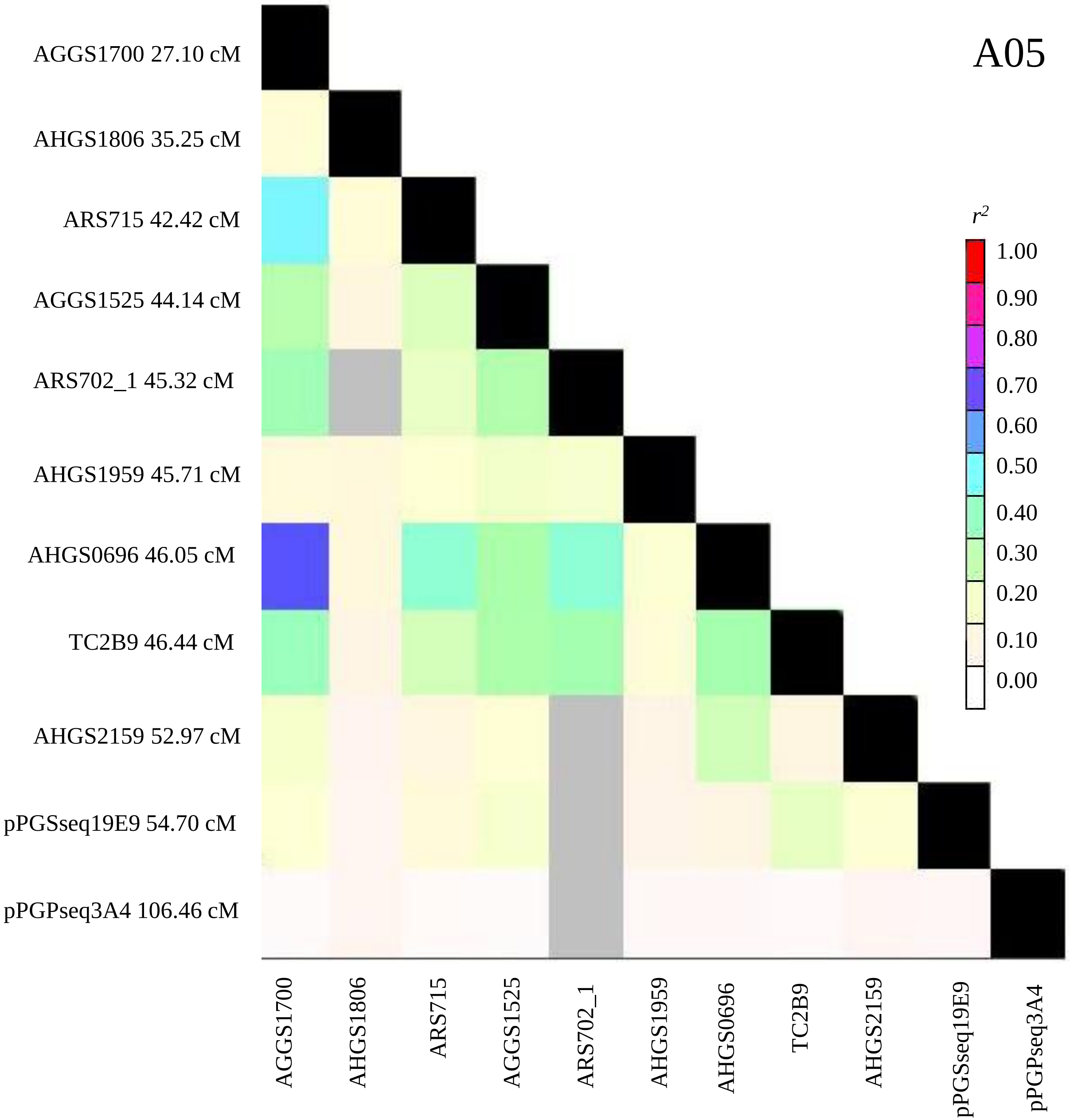
A05
AGGS1700 27.10 cM
AHGS1806 35.25 cM
ARS715 42.42 cM
AGGS1525 44.14 cM
ARS702_1 45.32 cM
AHGS1959 45.71 cM
AHGS0696 46.05 cM
TC2B9 46.44 cM
AHGS2159 52.97 cM
pPGSseq19E9 54.70 cM
pPGPseq3A4 106.46 cM
AGGS1700
AHGS1806
ARS715
AGGS1525
ARS702_1
AHGS1959
AHGS0696
TC2B9
AHGS2159
pPGSseq19E9
pPGPseq3A4
r^2
1.00
0.90
0.80
0.70
0.60
0.50
0.40
0.30
0.20
0.10
0.00

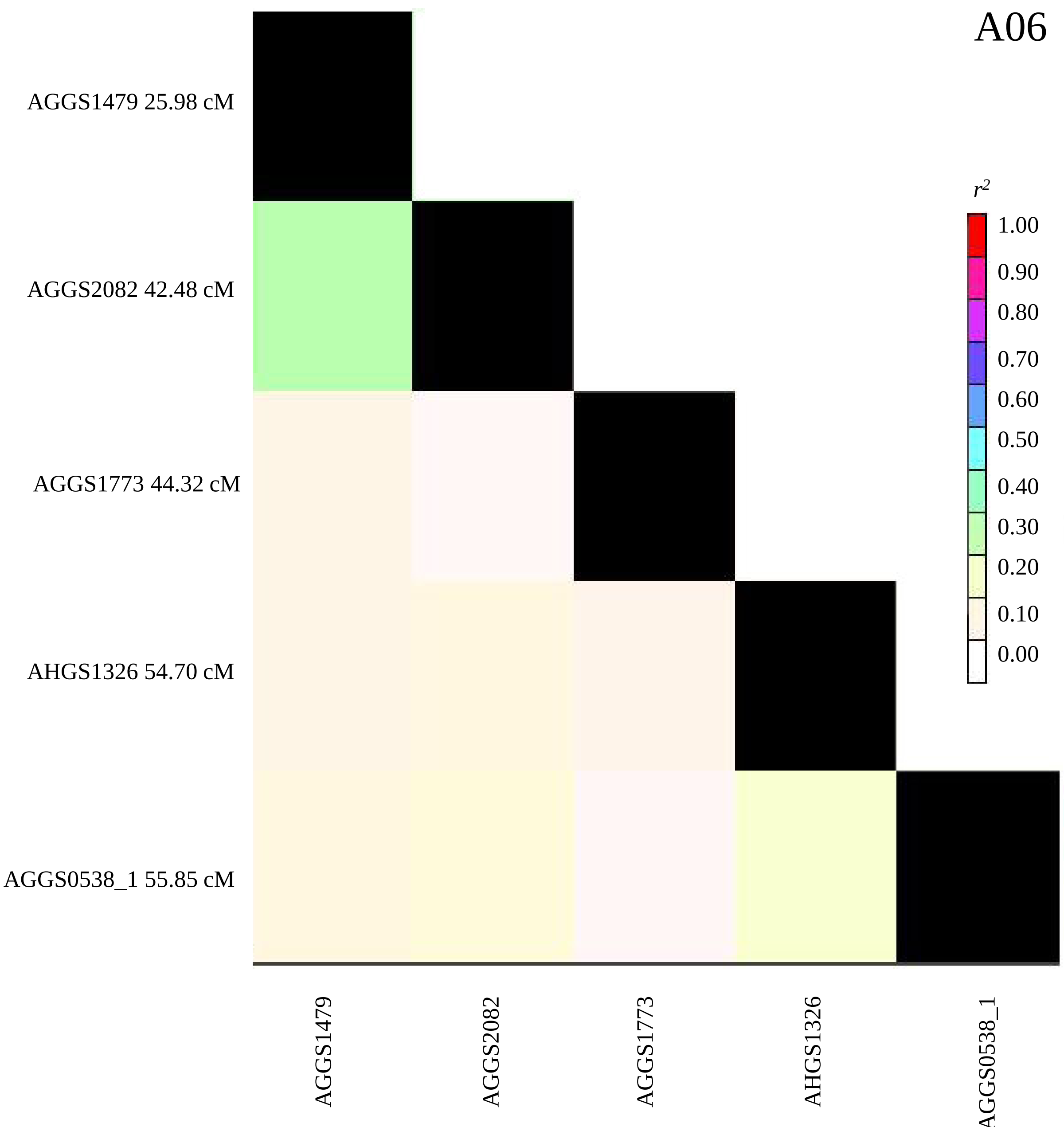

A06
AGGS1479 25.98 cM
AGGS2082 42.48 cM
AGGS1773 44.32 cM
AHGS1326 54.70 cM
AGGS0538_1 55.85 cM
r^2
1.00
0.90
0.80
0.70
0.60
0.50
0.40
0.30
0.20
0.10
0.00
AGGS1479
AGGS2082
AGGS1773
AHGS1326
AGGS0538_1

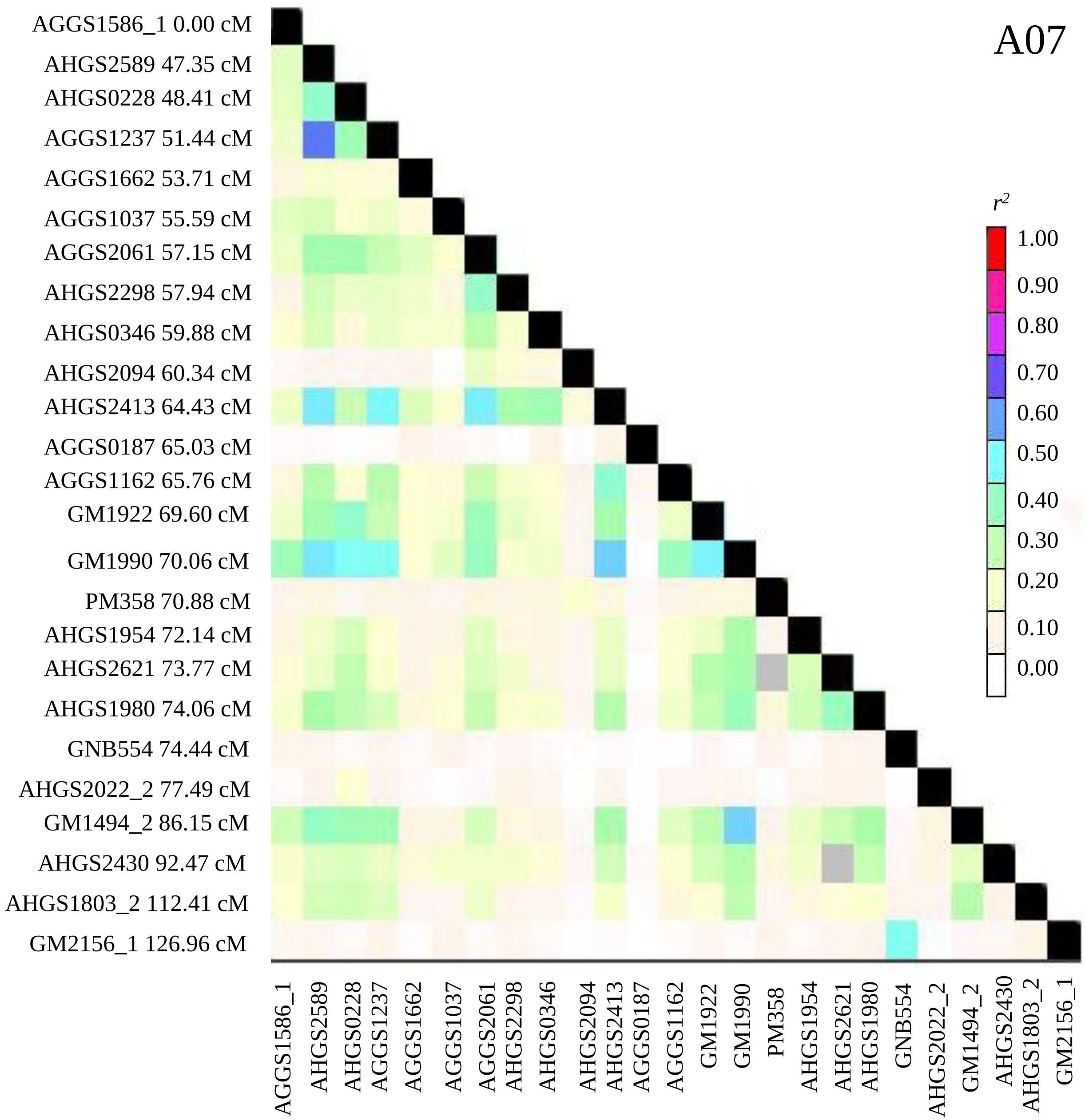
A07
AGGS1586_1 0.00 cM
AHGS2589 47.35 cM
AHGS0228 48.41 cM
AGGS1237 51.44 cM
AGGS1662 53.71 cM
AGGS1037 55.59 cM
AGGS2061 57.15 cM
AHGS2298 57.94 cM
AHGS0346 59.88 cM
AHGS2094 60.34 cM
AHGS2413 64.43 cM
AGGS0187 65.03 cM
AGGS1162 65.76 cM
GM1922 69.60 cM
GM1990 70.06 cM
PM358 70.88 cM
AHGS1954 72.14 cM
AHGS2621 73.77 cM
AHGS1980 74.06 cM
GNB554 74.44 cM
AHGS2022_2 77.49 cM
GM1494_2 86.15 cM
AHGS2430 92.47 cM
AHGS1803_2 112.41 cM
GM2156_1 126.96 cM
AGGS1586_1
AHGS2589
AHGS0228
AGGS1237
AGGS1662
AGGS1037
AGGS2061
AHGS2298
AHGS0346
AHGS2094
AHGS2413
AGGS0187
AGGS1162
GM1922
GM1990
PM358
AHGS1954
AHGS2621
AHGS1980
GNB554
AHGS2022_2
GM1494_2
AHGS2430
AHGS1803_2
GM2156_1
r2
1.00
0.90
0.80
0.70
0.60
0.50
0.40
0.30
0.20
0.10
0.00

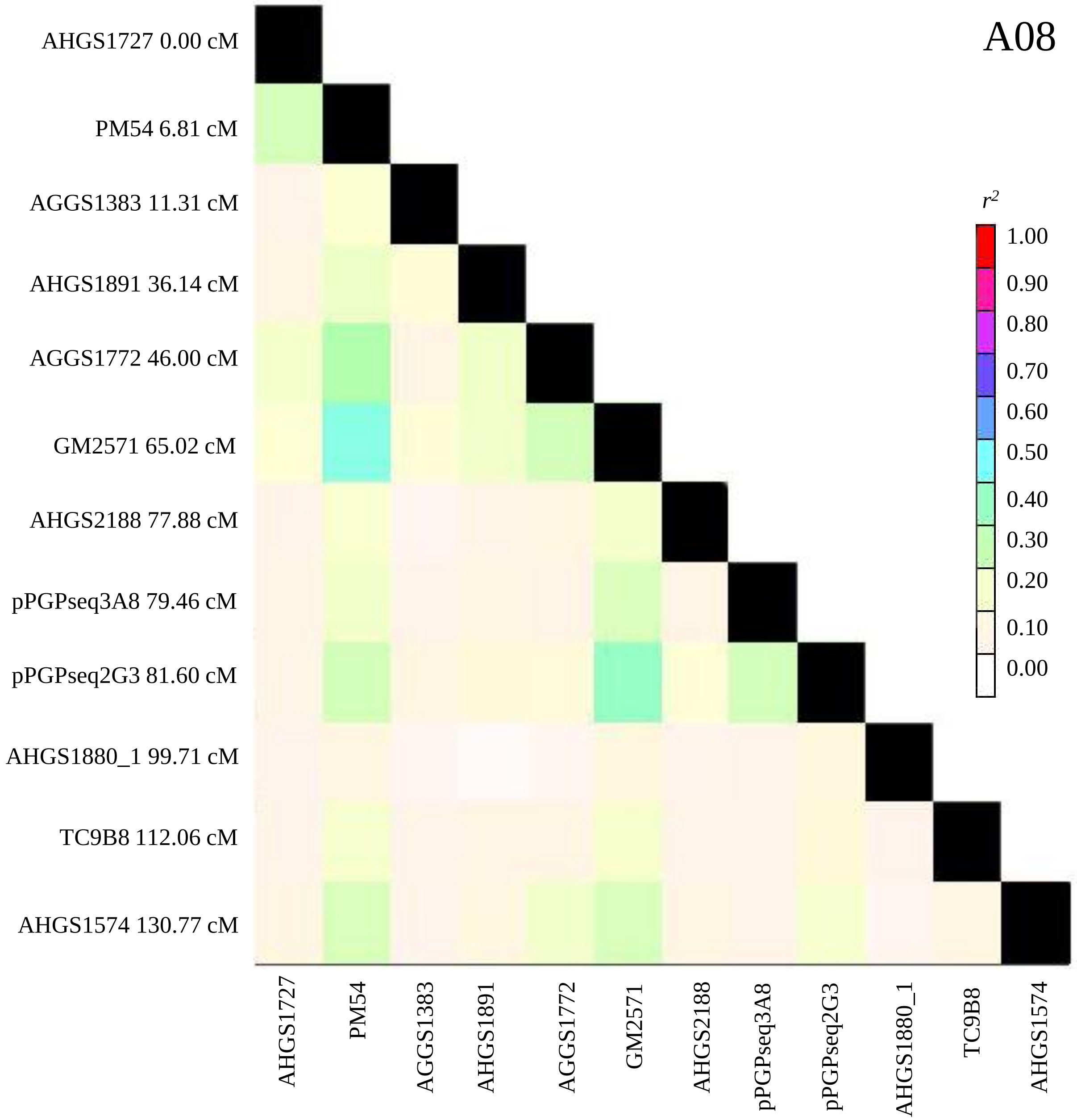

A08
AHGS1727 0.00 cM
PM54 6.81 cM
AGGS1383 11.31 cM
AHGS1891 36.14 cM
AGGS1772 46.00 cM
GM2571 65.02 cM
AHGS2188 77.88 cM
pPGPseq3A8 79.46 cM
pPGPseq2G3 81.60 cM
AHGS1880_1 99.71 cM
TC9B8 112.06 cM
AHGS1574 130.77 cM
AHGS1727
PM54
AGGS1383
AHGS1891
AGGS1772
GM2571
AHGS2188
pPGPseq3A8
pPGPseq2G3
AHGS1880_1
TC9B8
AHGS1574
r^2
1.00
0.90
0.80
0.70
0.60
0.50
0.40
0.30
0.20
0.10
0.00

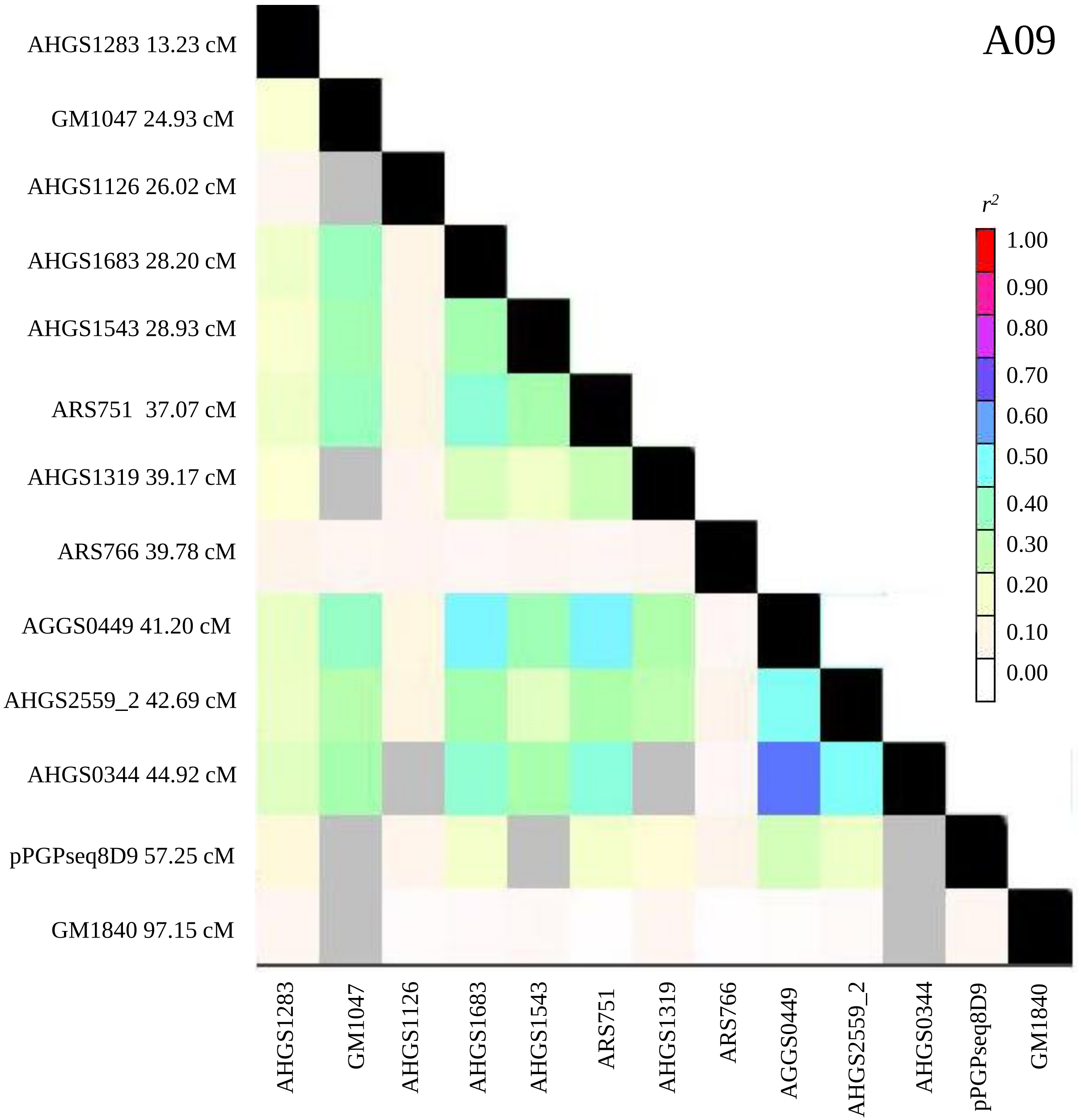

A09
AHGS1283 13.23 cM
GM1047 24.93 cM
AHGS1126 26.02 cM
AHGS1683 28.20 cM
AHGS1543 28.93 cM
ARS751 37.07 cM
AHGS1319 39.17 cM
ARS766 39.78 cM
AGGS0449 41.20 cM
AHGS2559_2 42.69 cM
AHGS0344 44.92 cM
pPGPseq8D9 57.25 cM
GM1840 97.15 cM
r²
1.00
0.90
0.80
0.70
0.60
0.50
0.40
0.30
0.20
0.10
0.00
AHGS1283
GM1047
AHGS1126
AHGS1683
AHGS1543
ARS751
AHGS1319
ARS766
AGGS0449
AHGS2559_2
AHGS0344
pPGPseq8D9
GM1840

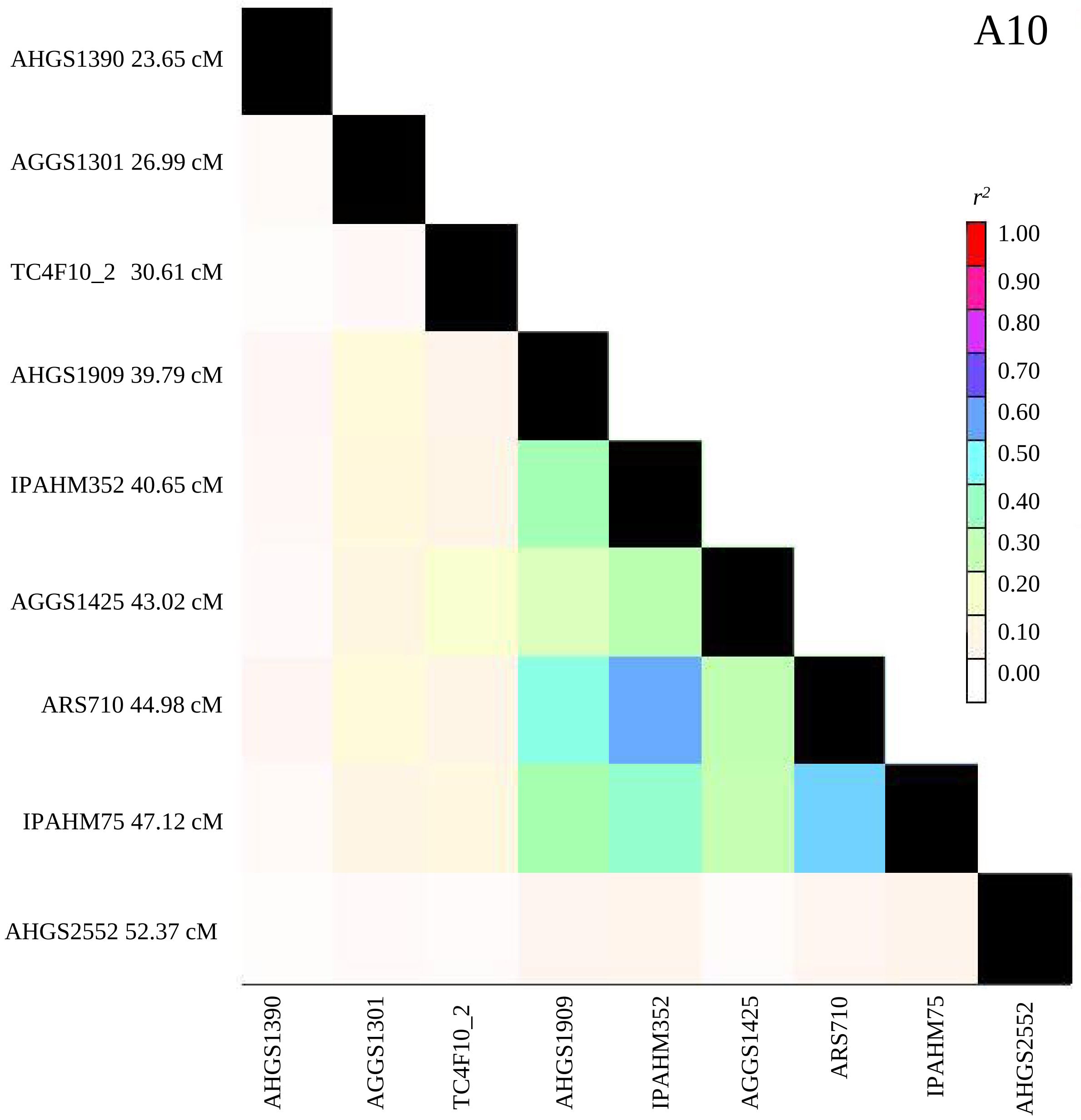
A10
AHGS1390 23.65 cM
AGGS1301 26.99 cM
TC4F10_2 30.61 cM
AHGS1909 39.79 cM
IPAHM352 40.65 cM
AGGS1425 43.02 cM
ARS710 44.98 cM
IPAHM75 47.12 cM
AHGS2552 52.37 cM
AHGS1390
AGGS1301
TC4F10_2
AHGS1909
IPAHM352
AGGS1425
ARS710
IPAHM75
AHGS2552
r²
1.00
0.90
0.80
0.70
0.60
0.50
0.40
0.30
0.20
0.10
0.00

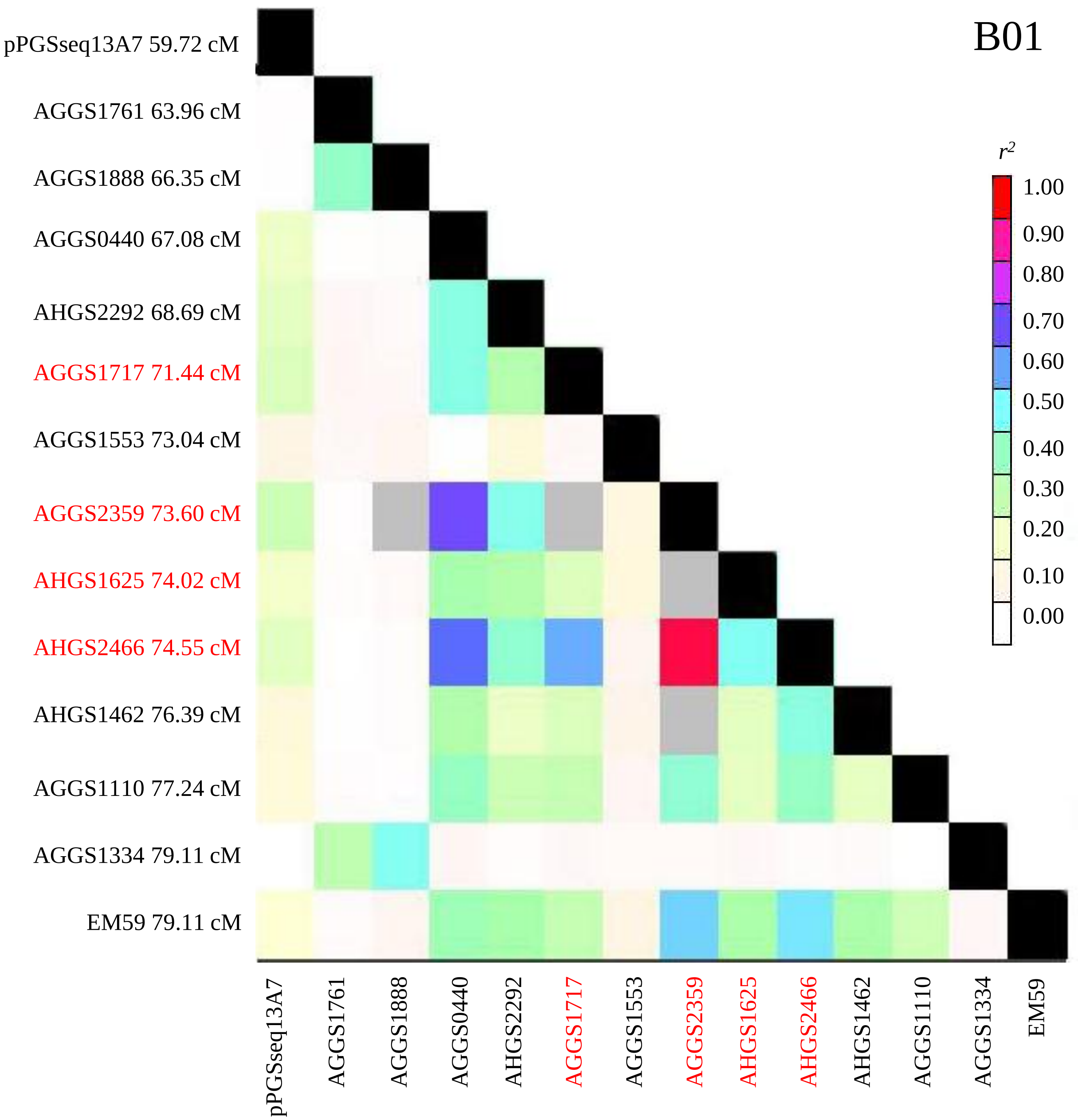
B01
pPGSseq13A7 59.72 cM
AGGS1761 63.96 cM
AGGS1888 66.35 cM
AGGS0440 67.08 cM
AHGS2292 68.69 cM
AGGS1717 71.44 cM
AGGS1553 73.04 cM
AGGS2359 73.60 cM
AHGS1625 74.02 cM
AHGS2466 74.55 cM
AHGS1462 76.39 cM
AGGS1110 77.24 cM
AGGS1334 79.11 cM
EM59 79.11 cM
r^2
1.00
0.90
0.80
0.70
0.60
0.50
0.40
0.30
0.20
0.10
0.00
pPGSseq13A7
AGGS1761
AGGS1888
AGGS0440
AHGS2292
AGGS1717
AGGS1553
AGGS2359
AHGS1625
AHGS2466
AHGS1462
AGGS1110
AGGS1334
EM59

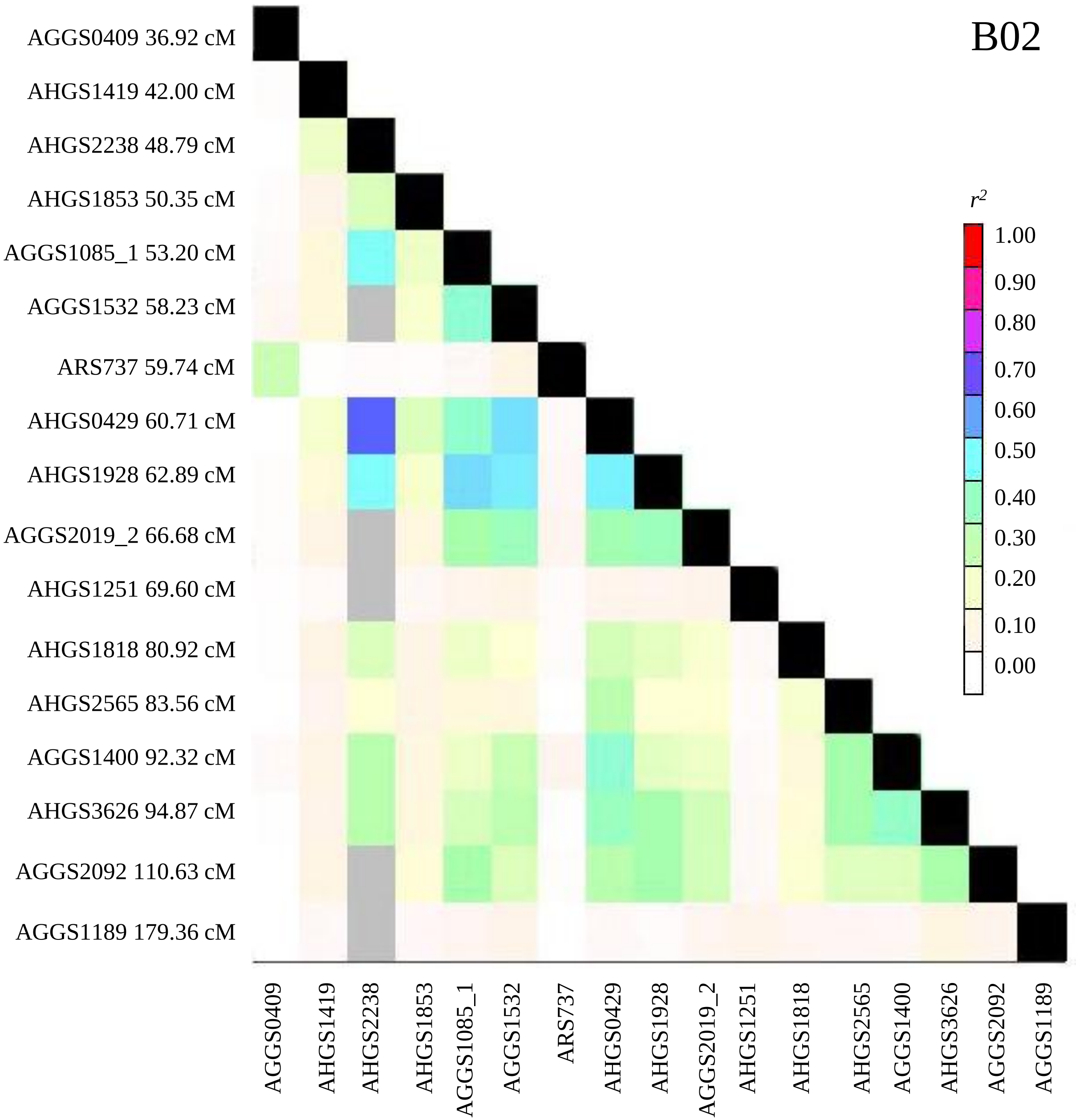
B02
AGGS0409 36.92 cM
AHGS1419 42.00 cM
AHGS2238 48.79 cM
AHGS1853 50.35 cM
AGGS1085_1 53.20 cM
AGGS1532 58.23 cM
ARS737 59.74 cM
AHGS0429 60.71 cM
AHGS1928 62.89 cM
AGGS2019_2 66.68 cM
AHGS1251 69.60 cM
AHGS1818 80.92 cM
AHGS2565 83.56 cM
AGGS1400 92.32 cM
AHGS3626 94.87 cM
AGGS2092 110.63 cM
AGGS1189 179.36 cM
AGGS0409
AHGS1419
AHGS2238
AHGS1853
AGGS1085_1
AGGS1532
ARS737
AHGS0429
AHGS1928
AGGS2019_2
AHGS1251
AHGS1818
AHGS2565
AGGS1400
AHGS3626
AGGS2092
AGGS1189
r2
1.00
0.90
0.80
0.70
0.60
0.50
0.40
0.30
0.20
0.10
0.00

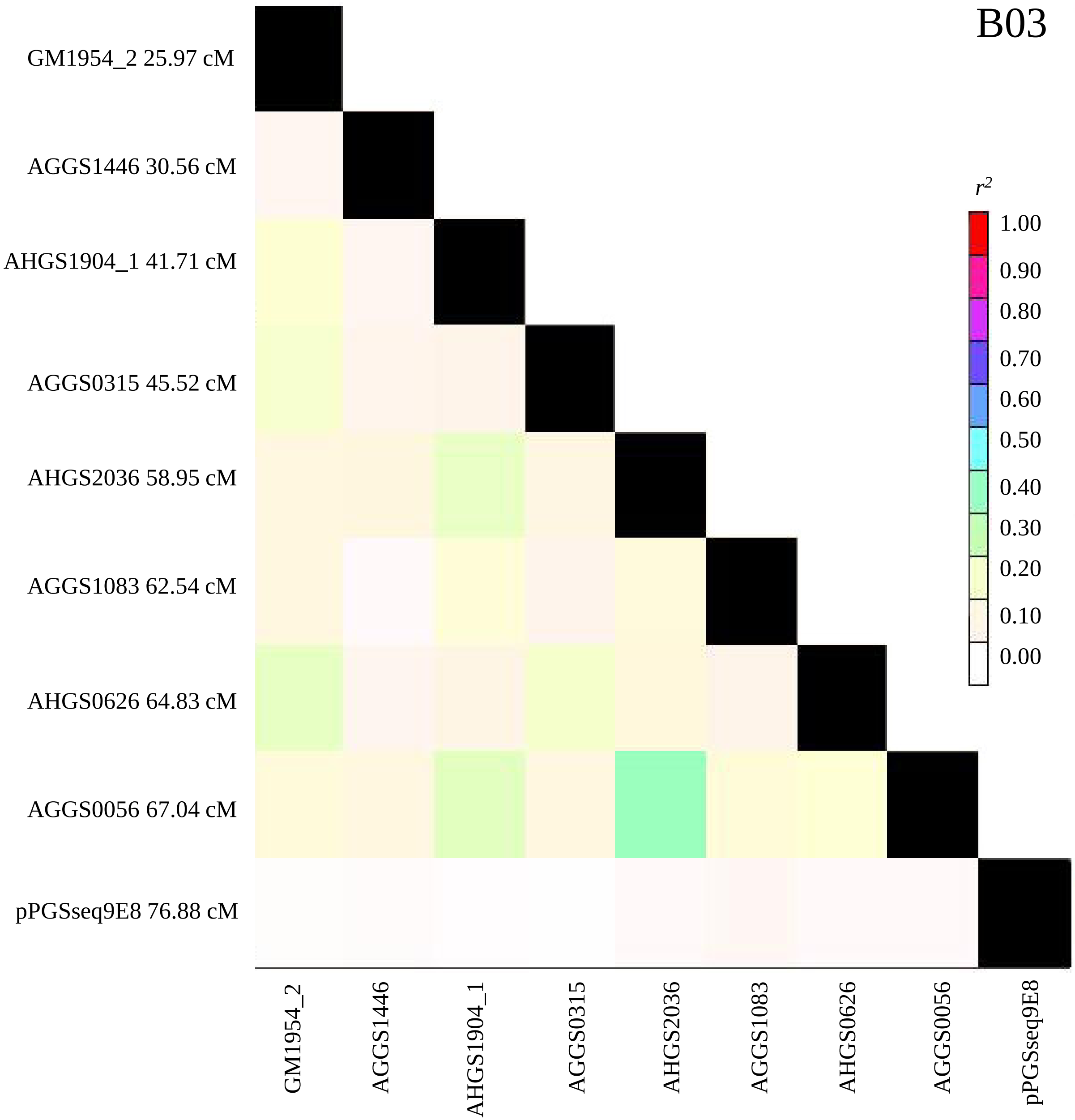
B03
GM1954_2 25.97 cM
AGGS1446 30.56 cM
AHGS1904_1 41.71 cM
AGGS0315 45.52 cM
AHGS2036 58.95 cM
AGGS1083 62.54 cM
AHGS0626 64.83 cM
AGGS0056 67.04 cM
pPGSseq9E8 76.88 cM
GM1954_2
AGGS1446
AHGS1904_1
AGGS0315
AHGS2036
AGGS1083
AHGS0626
AGGS0056
pPGSseq9E8
r^2
1.00
0.90
0.80
0.70
0.60
0.50
0.40
0.30
0.20
0.10
0.00

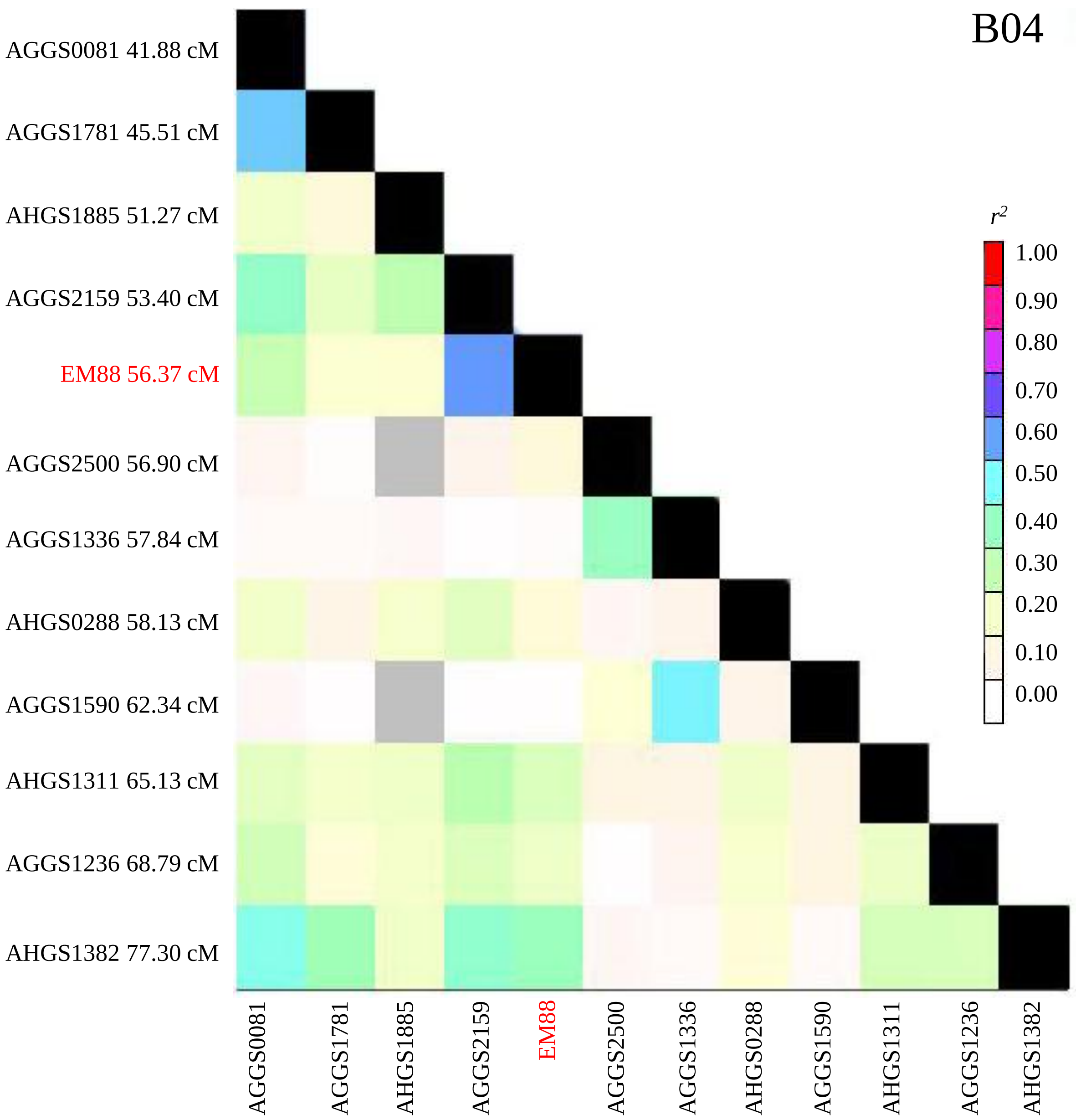
B04
AGGS0081 41.88 cM
AGGS1781 45.51 cM
AHGS1885 51.27 cM
AGGS2159 53.40 cM
EM88 56.37 cM
AGGS2500 56.90 cM
AGGS1336 57.84 cM
AHGS0288 58.13 cM
AGGS1590 62.34 cM
AHGS1311 65.13 cM
AGGS1236 68.79 cM
AHGS1382 77.30 cM
AGGS0081
AGGS1781
AHGS1885
AGGS2159
EM88
AGGS2500
AGGS1336
AHGS0288
AGGS1590
AHGS1311
AGGS1236
AHGS1382
r2
1.00
0.90
0.80
0.70
0.60
0.50
0.40
0.30
0.20
0.10
0.00

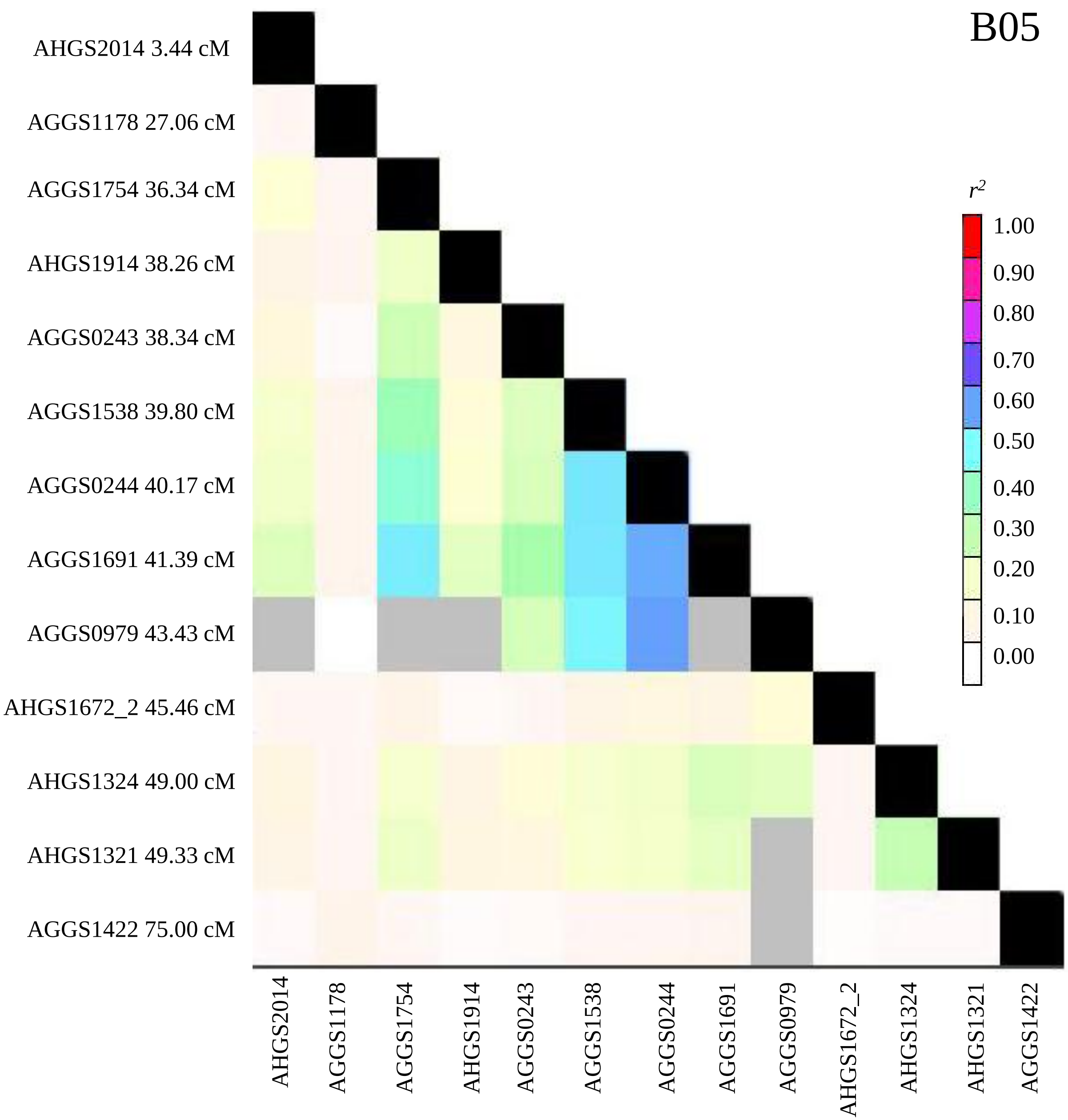
B05
AHGS2014 3.44 cM
AGGS1178 27.06 cM
AGGS1754 36.34 cM
AHGS1914 38.26 cM
AGGS0243 38.34 cM
AGGS1538 39.80 cM
AGGS0244 40.17 cM
AGGS1691 41.39 cM
AGGS0979 43.43 cM
AHGS1672_2 45.46 cM
AHGS1324 49.00 cM
AHGS1321 49.33 cM
AGGS1422 75.00 cM
AHGS2014
AGGS1178
AGGS1754
AHGS1914
AGGS0243
AGGS1538
AGGS0244
AGGS1691
AGGS0979
AHGS1672_2
AHGS1324
AHGS1321
AGGS1422
r2
1.00
0.90
0.80
0.70
0.60
0.50
0.40
0.30
0.20
0.10
0.00

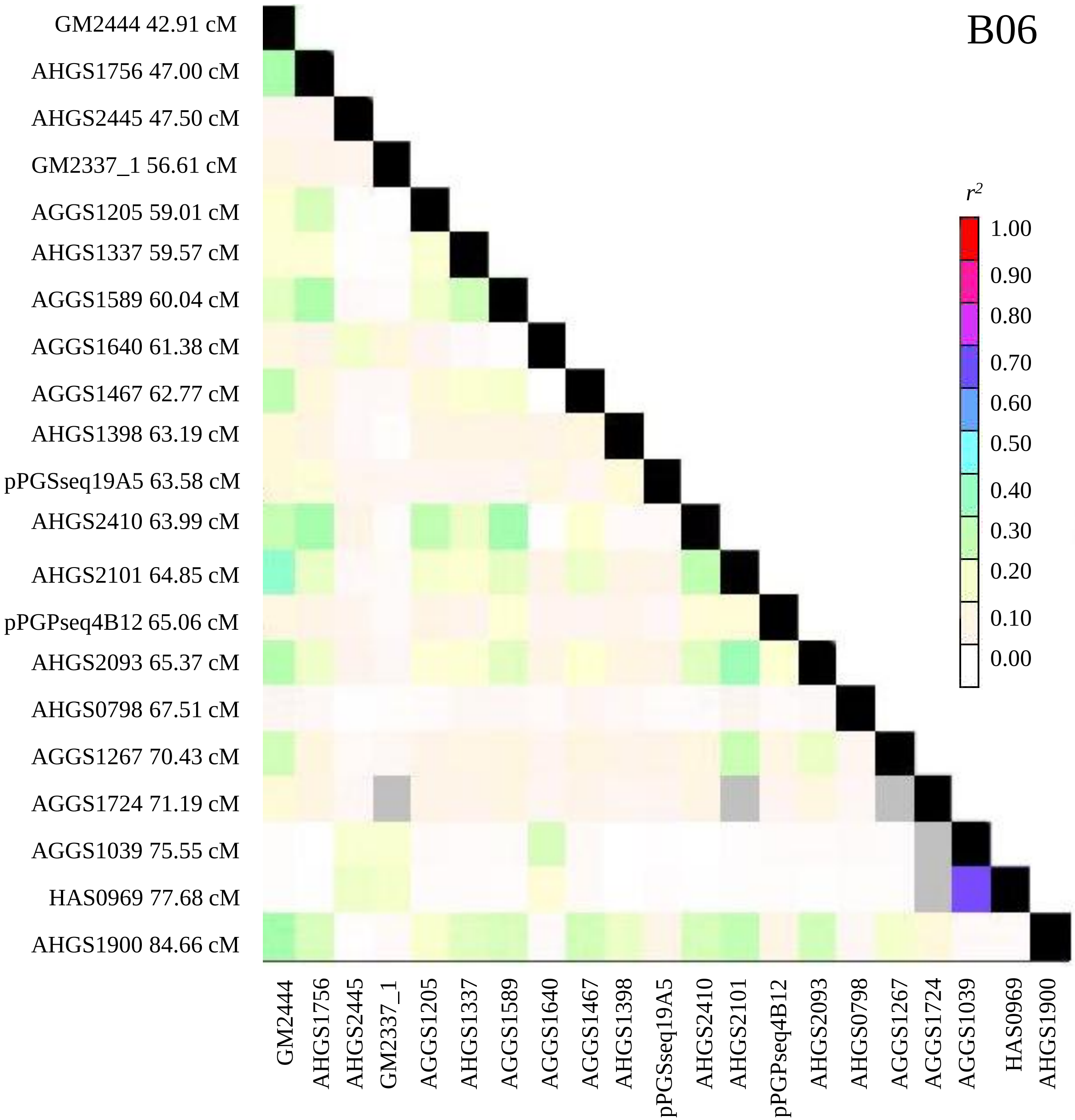
B06
GM2444 42.91 cM
AHGS1756 47.00 cM
AHGS2445 47.50 cM
GM2337_1 56.61 cM
AGGS1205 59.01 cM
AHGS1337 59.57 cM
AGGS1589 60.04 cM
AGGS1640 61.38 cM
AGGS1467 62.77 cM
AHGS1398 63.19 cM
pPGSseq19A5 63.58 cM
AHGS2410 63.99 cM
AHGS2101 64.85 cM
pPGPseq4B12 65.06 cM
AHGS2093 65.37 cM
AHGS0798 67.51 cM
AGGS1267 70.43 cM
AGGS1724 71.19 cM
AGGS1039 75.55 cM
HAS0969 77.68 cM
AHGS1900 84.66 cM
r^2
1.00
0.90
0.80
0.70
0.60
0.50
0.40
0.30
0.20
0.10
0.00
GM2444
AHGS1756
AHGS2445
GM2337_1
AGGS1205
AHGS1337
AGGS1589
AGGS1640
AGGS1467
AHGS1398
pPGSseq19A5
AHGS2410
AHGS2101
pPGPseq4B12
AHGS2093
AHGS0798
AGGS1267
AGGS1724
AGGS1039
HAS0969
AHGS1900

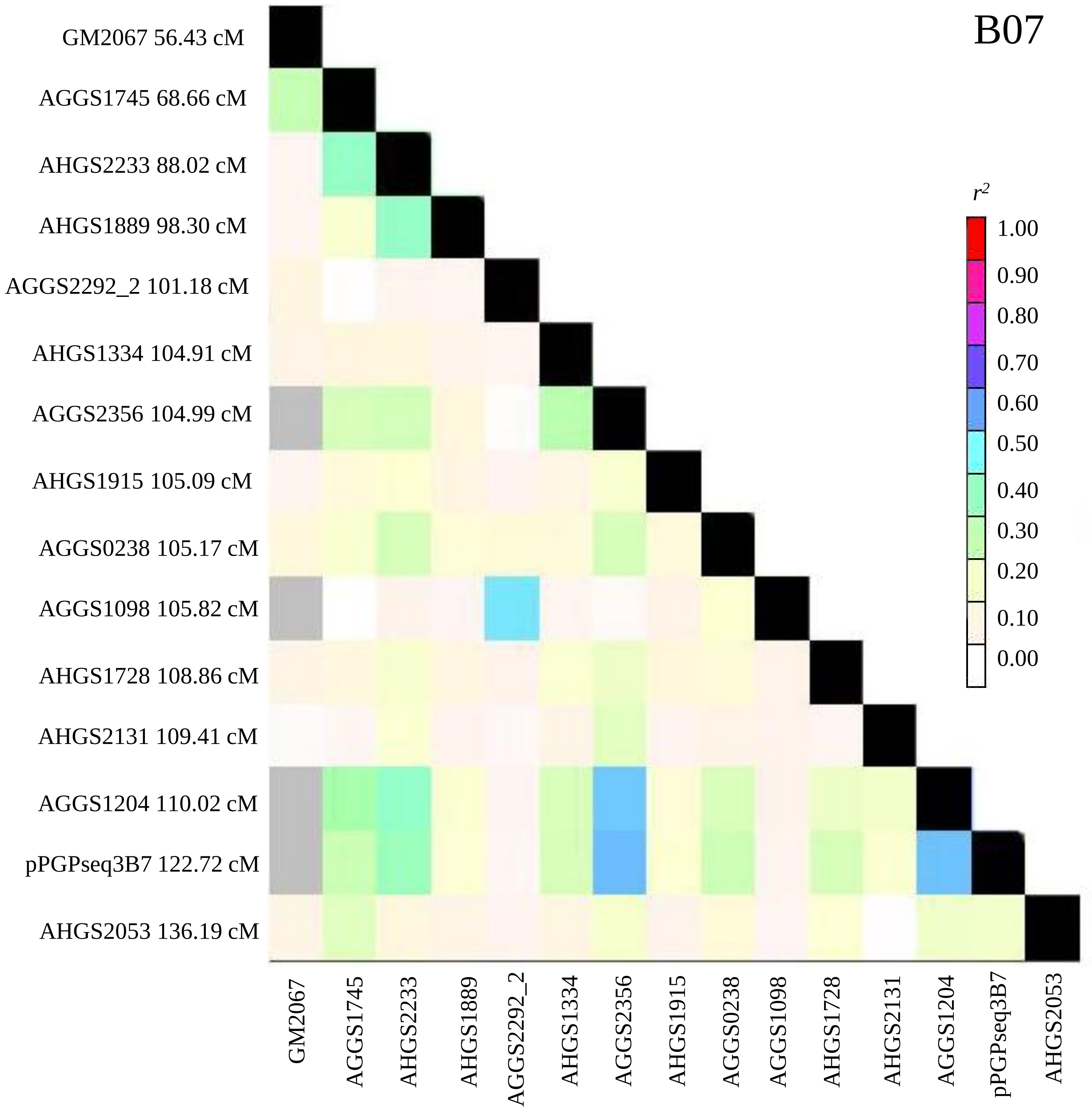
B07
GM2067 56.43 cM
AGGS1745 68.66 cM
AHGS2233 88.02 cM
AHGS1889 98.30 cM
AGGS2292_2 101.18 cM
AHGS1334 104.91 cM
AGGS2356 104.99 cM
AHGS1915 105.09 cM
AGGS0238 105.17 cM
AGGS1098 105.82 cM
AHGS1728 108.86 cM
AHGS2131 109.41 cM
AGGS1204 110.02 cM
pPGPseq3B7 122.72 cM
AHGS2053 136.19 cM
r2
1.00
0.90
0.80
0.70
0.60
0.50
0.40
0.30
0.20
0.10
0.00
GM2067
AGGS1745
AHGS2233
AHGS1889
AGGS2292_2
AHGS1334
AGGS2356
AHGS1915
AGGS0238
AGGS1098
AHGS1728
AHGS2131
AGGS1204
pPGPseq3B7
AHGS2053

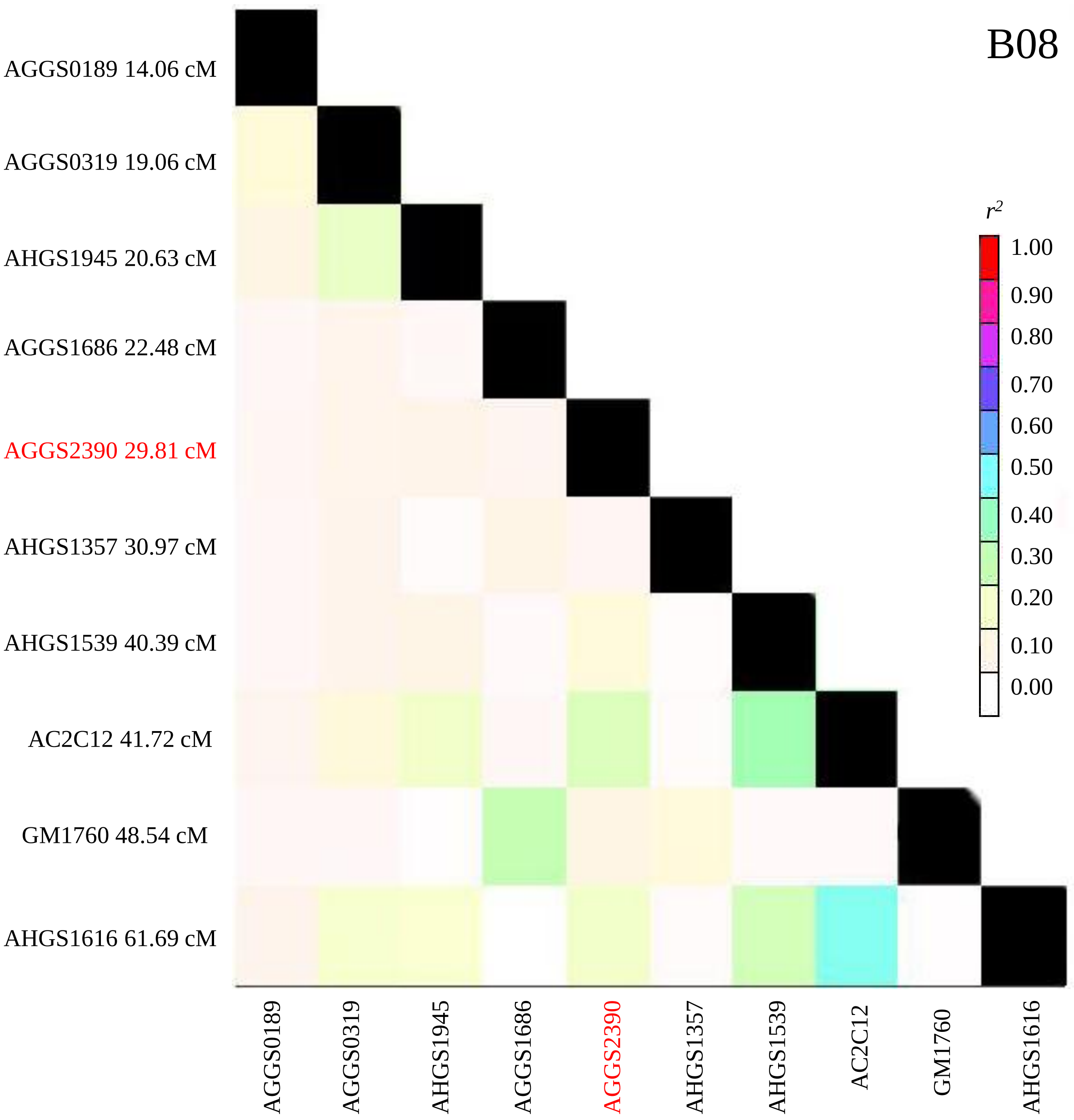
B08
AGGS0189 14.06 cM
AGGS0319 19.06 cM
AHGS1945 20.63 cM
AGGS1686 22.48 cM
AGGS2390 29.81 cM
AHGS1357 30.97 cM
AHGS1539 40.39 cM
AC2C12 41.72 cM
GM1760 48.54 cM
AHGS1616 61.69 cM
AGGS0189
AGGS0319
AHGS1945
AGGS1686
AGGS2390
AHGS1357
AHGS1539
AC2C12
GM1760
AHGS1616
r^2
1.00
0.90
0.80
0.70
0.60
0.50
0.40
0.30
0.20
0.10
0.00

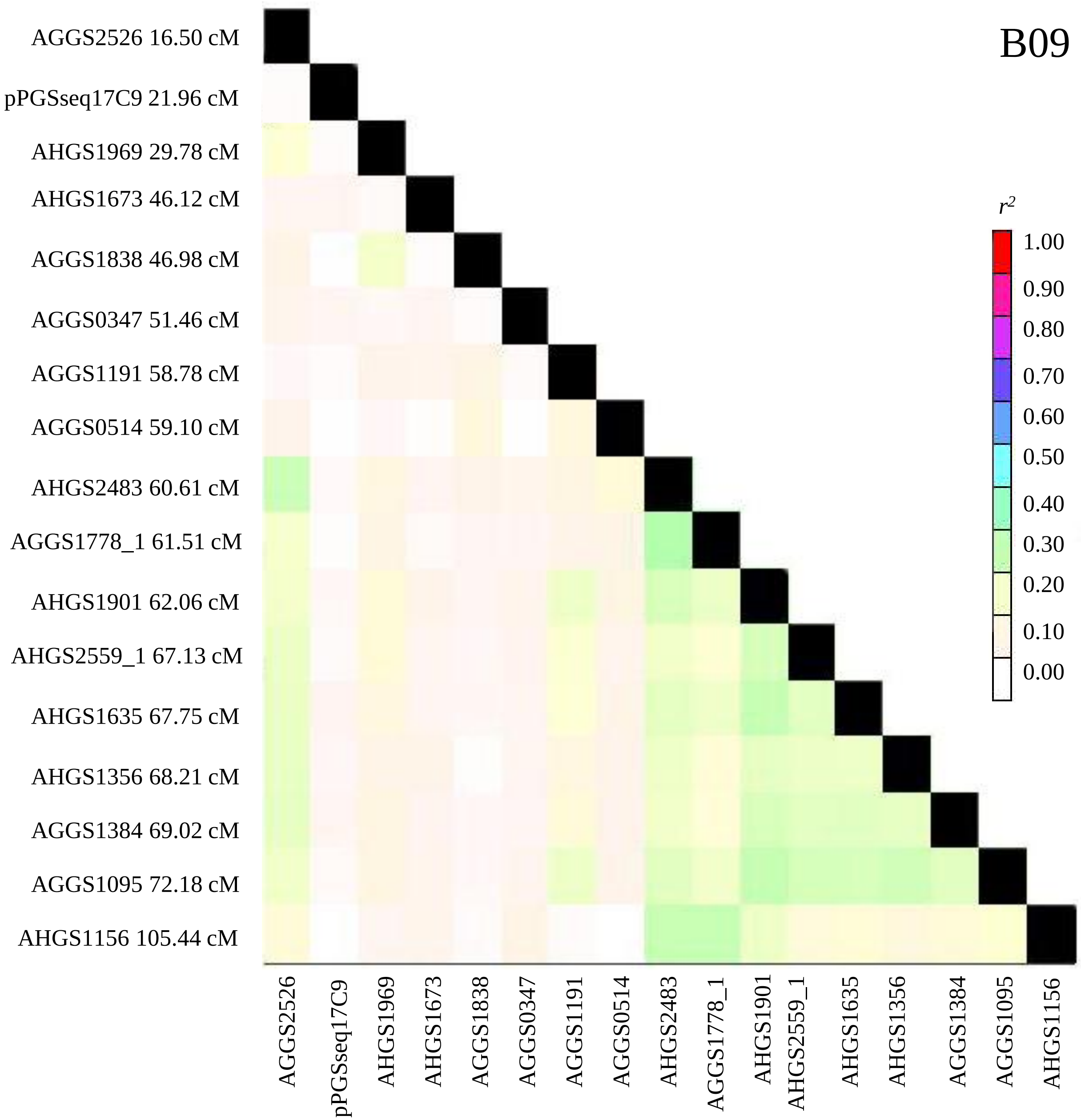
B09
AGGS2526 16.50 cM
pPGSseq17C9 21.96 cM
AHGS1969 29.78 cM
AHGS1673 46.12 cM
AGGS1838 46.98 cM
AGGS0347 51.46 cM
AGGS1191 58.78 cM
AGGS0514 59.10 cM
AHGS2483 60.61 cM
AGGS1778_1 61.51 cM
AHGS1901 62.06 cM
AHGS2559_1 67.13 cM
AHGS1635 67.75 cM
AHGS1356 68.21 cM
AGGS1384 69.02 cM
AGGS1095 72.18 cM
AHGS1156 105.44 cM
AGGS2526
pPGSseq17C9
AHGS1969
AHGS1673
AGGS1838
AGGS0347
AGGS1191
AGGS0514
AHGS2483
AGGS1778_1
AHGS1901
AHGS2559_1
AHGS1635
AHGS1356
AGGS1384
AGGS1095
AHGS1156
r^2
1.00
0.90
0.80
0.70
0.60
0.50
0.40
0.30
0.20
0.10
0.00

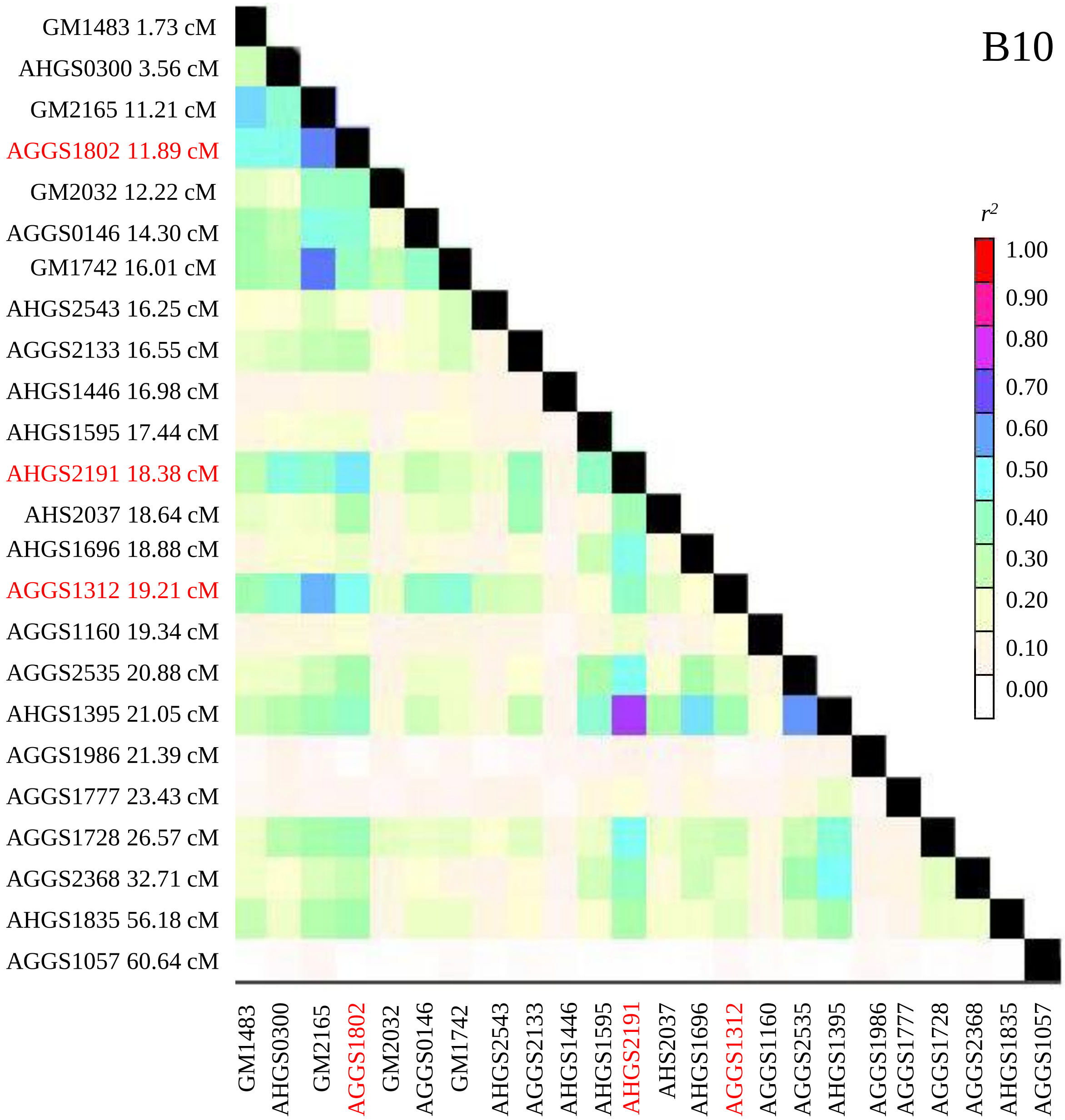
B10
GM1483 1.73 cM
AHGS0300 3.56 cM
GM2165 11.21 cM
AGGS1802 11.89 cM
GM2032 12.22 cM
AGGS0146 14.30 cM
GM1742 16.01 cM
AHGS2543 16.25 cM
AGGS2133 16.55 cM
AHGS1446 16.98 cM
AHGS1595 17.44 cM
AHGS2191 18.38 cM
AHS2037 18.64 cM
AHGS1696 18.88 cM
AGGS1312 19.21 cM
AGGS1160 19.34 cM
AGGS2535 20.88 cM
AHGS1395 21.05 cM
AGGS1986 21.39 cM
AGGS1777 23.43 cM
AGGS1728 26.57 cM
AGGS2368 32.71 cM
AHGS1835 56.18 cM
AGGS1057 60.64 cM
GM1483
AHGS0300
GM2165
AGGS1802
GM2032
AGGS0146
GM1742
AHGS2543
AGGS2133
AHGS1446
AHGS1595
AHGS2191
AHS2037
AHGS1696
AGGS1312
AGGS1160
AGGS2535
AHGS1395
AGGS1986
AGGS1777
AGGS1728
AGGS2368
AHGS1835
AGGS1057
r^2
1.00
0.90
0.80
0.70
0.60
0.50
0.40
0.30
0.20
0.10
0.00
